# Doubly charged dimers and trimers of heavy noble gases†

**DOI:** 10.1039/d4cp00465e

**Published:** 2024-03-23

**Authors:** Gabriel Schöpfer, Stefan Bergmeister, Milan Ončák, Ianessa Stromberg, Masoomeh Mahmoodi-Darian, Paul Scheier, Olof Echt, Elisabeth Gruber

**Affiliations:** a Institut für Ionenphysik und Angewandte Physik, Universität Innsbruck Innsbruck Austria Milan.Oncak@uibk.ac.at Olof.Echt@unh.edu; b School of Chemistry, University of Edinburgh Edinburgh UK; c Department of Physics, University of New Hampshire Durham USA

## Abstract

Many doubly charged heteronuclear dimers are metastable or even thermodynamically stable with respect to charge separation. Homonuclear dicationic dimers, however, are more difficult to form. He_2_^2+^ was the first noble gas dimer predicted to be metastable and, decades later, observed. Ne_2_^2+^ is the only other dicationic noble gas dimer that has been detected so far. Here, we present a novel approach to form fragile dicationic species, by post-ionization of singly charged ions that are embedded in helium nanodroplets (HNDs). Bare ions are then extracted by colliding the HNDs with helium gas. We detect homonuclear doubly charged dimers and trimers of krypton and xenon, but not argon. Our multi-reference *ab initio* calculations confirm the stability of Kr_2_^2+^, Kr_3_^2+^, Xe_2_^2+^, Xe_3_^2+^, and Ar_2_^2+^, but put the stability of Ar_3_^2+^ towards dissociation to Ar^+^ + Ar_2_^+^ into question.

## Abstract 2

Long-lived doubly charged dimers and trimers of krypton and xenon are formed by sequential ionization of doped helium nanodroplets.

## Graphical abstract

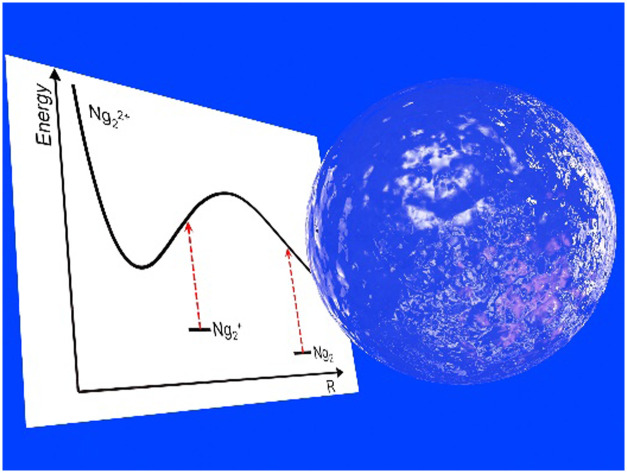

## 1 Introduction

In 1933, Pauling postulated the existence of metastable He_2_^2+^.^1^ He calculated that the local minimum lies more than 10 eV above the separated He^+^ + He^+^ ion pair at an internuclear distance of 0.75 Å and supports 4 vibrational states. It took five decades until He_2_^2+^ could be prepared by charge stripping of He_2_^+^.^2,3^ The observation was indirect, only showing the He^+^ fragments, but in later experiments He_2_^2+^ with a lifetime exceeding 5 μs could be identified.^4^

A few years later, Ne_2_^2+^ was detected in charge-stripping collisions of Ne_2_^+^ with Ar, and by field desorption.^5,6^ A lifetime of at least 1 μs was inferred. The results were puzzling because theoretical work shows that the ^1^Σ^+^_g_ ground state of Ne_2_^2+^ is purely repulsive.^7–9^

Several experimental and theoretical reports have been devoted to the heavier noble gases (Ng).^10^ Argon clusters are a model system for the study of interatomic coulombic decay (ICD). When a core electron is removed from an atom in Ar_2_, the hole may be filled by a valence electron and the excess energy causes the emission of another valence electron (a so-called Auger–Meitner electron), resulting in Ar–Ar^2+^. Alternatively, in the ICD process,^11,12^ the energy released upon filling the hole is transferred to a valence electron in the other atom, resulting in Ar^+^–Ar^+^.

ICD in argon clusters has been investigated by measuring the kinetic energy of the emitted electrons, ions, and by electron–ion and ion–ion coincidence experiments. The presence of a fleeting Ar_2_^2+^ has been inferred from the observation of high-energy fragment ions but intact Ar_2_^2+^ has never been detected.^12–20^ Ar_2_^2+^ has a local minimum in the potential energy curve (PEC) at about 2 Å but this quasi-bound region cannot be reached by vertical ionization of Ar_2_ which has an internuclear distance of 3.76 Å.^21–23^

The processes following core-level excitation in krypton and xenon clusters have been studied by similar techniques.^23–27^ Again, the existence of transient dicationic dimers Ng_2_^2+^ has been inferred from the data but intact Ng_2_^2+^ was not observed. Doubly charged noble gas dimers are also of interest because they may give rise to the so-called third excimer continuum in the VUV emission spectra of the noble gases.^27^

Here we explore a novel method to form dications of van der Waals bound systems. As shown recently, fragile ionic species such as SF_6_^+^ or the phenanthrene anion Ph^−^ can be synthesized in and gently extracted from helium nanodroplets (HNDs).^28–30^ We have added a second ionizer to the experimental apparatus, making it possible to form dications by sequential ionization.^31^ Vertical ionization of a singly-charged molecular ion offers a much better chance to reach the metastable dicationic state than vertical ionization of a neutral molecule.^21^

With this novel approach^31^ we are able to detect long-lived Kr_2_^2+^ and Xe_2_^2+^ in a high-resolution mass spectrometer. We also observe doubly-charged trimers, but no tetramers. Similar experiments with argon do not produce small dicationic species but this failure may be due to technical challenges. Our experiments are supported by *ab initio* calculations that quantify the respective Coulomb barriers and confirm Ar_2_^2+^ as a metastable ion but predict Ar_3_^2+^ to dissociate spontaneously into Ar_2_^+^ + Ar^+^.

## 2 Methods

### 2.1 Experimental details

An earlier version of the apparatus^32^ was recently upgraded by the implementation of a second electron impact ionizer.^31^ HNDs containing some 10^6^ atoms are produced in an expansion of helium gas through a 5 μm nozzle into vacuum. Interaction with an intense 40 eV electron beam introduces a few to a few dozen positive charges into the HND,^33^ resulting in He_*N*_^*z*+^ nanodroplets that contain *z* molecular helium ions, probably He_3_^+^.^34,35^ The ions reside near the surface of the HND because of mutual coulombic repulsion.^36^ The He_*N*_^*z*+^ nanodroplets are passed through an electrostatic deflector which selects them by their mass-to-charge ratio, hence their size-to-charge ratio *N*/*z*, and are then guided through a pickup cell filled with a low-density noble gas. Captured Ng atoms agglomerate in the HNDs at the charge centers and form *z* singly charged clusters Ng_*n*_^+^.

The ion beam is intersected by another intense electron beam at 40 eV which introduces additional charges in the droplets. It will also electronically excite helium. The electronic excitation may find its way to the dopant and Penning ionize Ng_*n*_^+^ to Ng_*n*_^2+^.^35,37–39^ The HNDs are then collided with helium gas in an ion guide, leading to the gentle escape of the dopant ions from the droplet. Their mass-to-charge ratio *m*/*z* is determined in a time-of-flight mass spectrometer (TOF-MS) equipped with an electrostatic reflectron. The mass resolution is about 10^4^ full-width at half-maximum.

### 2.2 Computational details

**Scheme 1:**
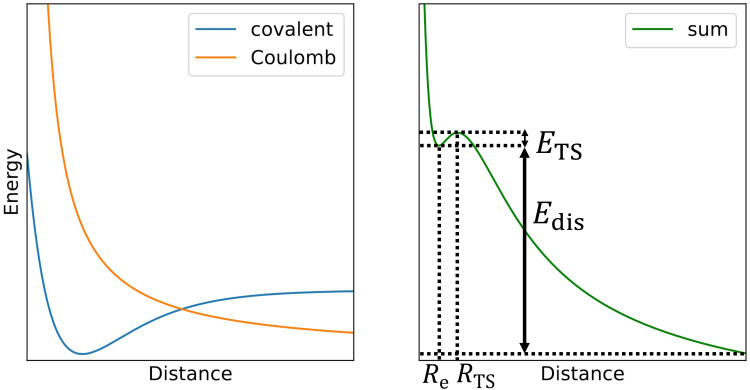
In the first approximation, the potential energy curve for dissociation of doubly-charge ions (right) can be thought as a sum of a covalent bond potential and Coulomb repulsion (left).

The challenge in computational treatment of doubly-charged rare gas dimers and trimers is the metastability evolving from the competition between the covalent bond formed at short distances and the Coulomb repulsion between nuclei destabilizing the system (Scheme 1). Here, we use both single-reference and multi-reference methods to describe the electronic structure of the metastable ions. Namely, we employ the coupled cluster singles and doubles with non-iteratively included triplets (CCSD(T)) and multi-reference configuration interaction singles and doubles (MRCI) based on complete active space self-consistent field (CASSCF). Single-reference CCSD(T) calculations are employed for calculating the dissociation energy while MRCI is used to describe the Coulomb barrier. Both methods are used to predict the metastable minima.

The active space size is a crucial parameter in multi-reference calculations. Detailed active space benchmarking, orbital composition of various active spaces and included electronic states can be found in the ESI,† Fig. S2–S5 and Tables S1–S4. Here, we only summarize the respective conclusions. The Ng_2_^2+^ and Ng_3_^2+^ ions are calculated as linear, corresponding to *D*_∞h_ and *C*_∞v_ point groups lowered to *D*_2h_ and *C*_2v_ point groups, respectively, in actual MRCI calculations. For Ng_2_^2+^, we included six orbitals correlated to valence p orbitals in Ng^+^ (σ^+^_g_, σ^+^_u_, π_g_, π_u_) with 10 electrons, and gradually increased the active space by including more virtual orbitals, finally employing 10 electrons in 11 orbitals, (10,11). For the full electronic state analysis of Ar_2_^2+^, we included all 36 states which correlate with the Ar (^2^P) + Ar (^2^P) asymptote, namely 9 singlet (2x ^1^Σ^+^_g_, ^1^Π_g_, ^1^Δ_g_, ^1^Σ^−^_u_, ^1^Π_u_) and 27 triplet (^3^Σ^−^_g_, ^3^Π_g_, 2x ^3^Σ^+^_u_, ^3^Π_u_, ^3^Δ_u_) electronic states. For the scans, we included 5 singlet (^1^Σ^+^_g_, ^1^Π_g_, ^1^Π_u_) and 12 triplet (^3^Π_g_, ^3^Π_u_) electronic states. In Ng_3_^2+^, we included 16 electrons in 9 valence orbitals (σ^+^_g_, 2x σ^+^_u_, π_g_, 2x π_u_), again enlarging the active space with further virtual orbitals in the second step. In the main text, results of (16,9) and (16,10) active spaces are compared. We included 5 singlet (^1^Σ^+^_g_, ^1^Π_g_, ^1^Π_u_) and 15 triplet (^3^Π_g_, ^3^Σ^+^_u_, ^3^Π_u_) electronic states. For the 2D scans in the lower *C*_∞v_ symmetry, we used the orbitals and electronic states corresponding to the ones in the *D*_∞h_ calculations. We note that empirical Davidson correction to estimate the influence of higher-order excitations influences somewhat the dissociation barriers as shown in the ESI† (Fig. S2, S3 and S5).

The zero-point correction in dissociation energies is included as calculated at the CCSD level, no zero-point correction is used for calculating Coulomb barriers. Within single-reference calculations, restricted Hartree–Fock (HF) wave functions are used for singlet species. In Ng_3_^2+^ ions, stabilization of the electronic wave function in the HF calculation leads to an unrestricted wave function with lower HF and CCSD energies, predicting no metastable minimum for Ar_3_^2+^ and Kr_3_^2+^. This further emphasizes the complex electronic structure of the species. In all calculations, the def2QZVPPD basis set is employed. The Gaussian software is used for single-reference calculations,^40^ Molpro for multi-reference ones.^41,42^

## 3 Results

**Fig. 1:**
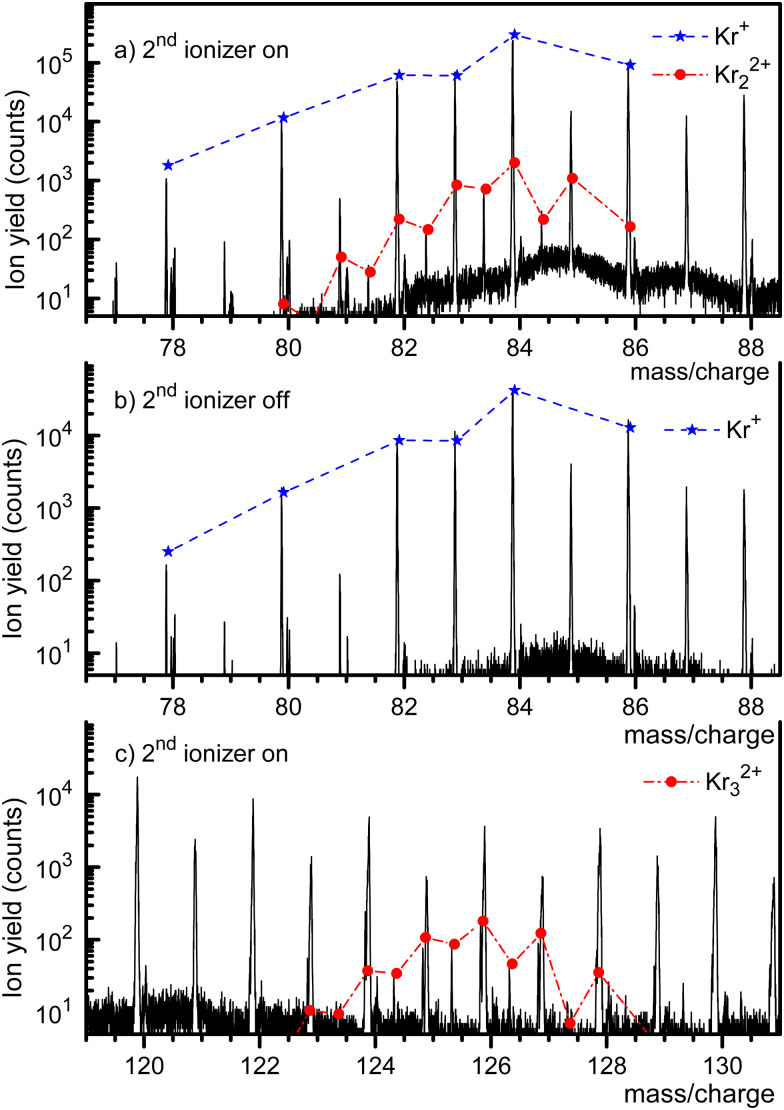
A mass spectrum of HNDs doped with Kr reveals the existence of Kr_2_^2+^ and Kr_3_^2+^ when both ionizers are turned on (panels a and c, respectively). The expected abundance distributions of their isotopologues are indicated by dash-dotted lines. Only singly charged Kr ions are seen when the second ionizer is turned off (panel b).

Fig. 1 displays a mass spectrum of HNDs doped with Kr. The prominent mass peaks in panel a (recorded with both ionizers turned on, note the logarithmic *y*-scale) are due to Kr isotopes ^78^Kr^+^ through ^86^Kr^+^. Their natural abundance distribution is indicated by the dashed line. The line describes the observed ion yield quite well. Mass peaks due to Kr_2_^2+^ with an even mass number cannot be distinguished from those due to singly charged monomers, but they can be identified if their nominal mass is an odd integer. Mass peaks due to ^80^Kr^83^Kr^2+^, ^82^Kr^83^Kr^2+^, ^84^Kr^83^Kr^2+^, and ^86^Kr^83^Kr^2+^ are, indeed, clearly seen. The expected abundance distribution of the isotopologues of Kr_2_^2+^, indicated by the dash-dotted line, agrees well with the data when *m*/*z* is half-integer.

The mass spectrum in Fig. 1b covers the same *m*/*z* range as in Fig. 1a. It was recorded with the second ionizer turned off. As expected, no ions appear at half-integer *m*/*z* values. The spectrum in Fig. 1c (recorded with both ionizers on) covers the range where doubly charged trimers would appear. Indeed, we observe three mass peaks at half-integer *m*/*z* values. Each of these peaks has contributions from several isotopologues whose exact *m*/*z* values differ by less than ≈0.002. The individual peaks cannot be resolved, but the expected cumulative abundance distribution describes the measured peak heights well. The half-integer mass peaks in Fig. 1c disappear when the second ionizer is turned off, see the ESI† (Fig. S1). The prominent mass peaks in Fig. 1c at integer *m*/*z* are mostly due to He_*m*_Kr^+^. These complexes also account for the strong mass peaks at *m*/*z* = 87 and 88 in Fig. 1a and b.

**Fig. 2:**
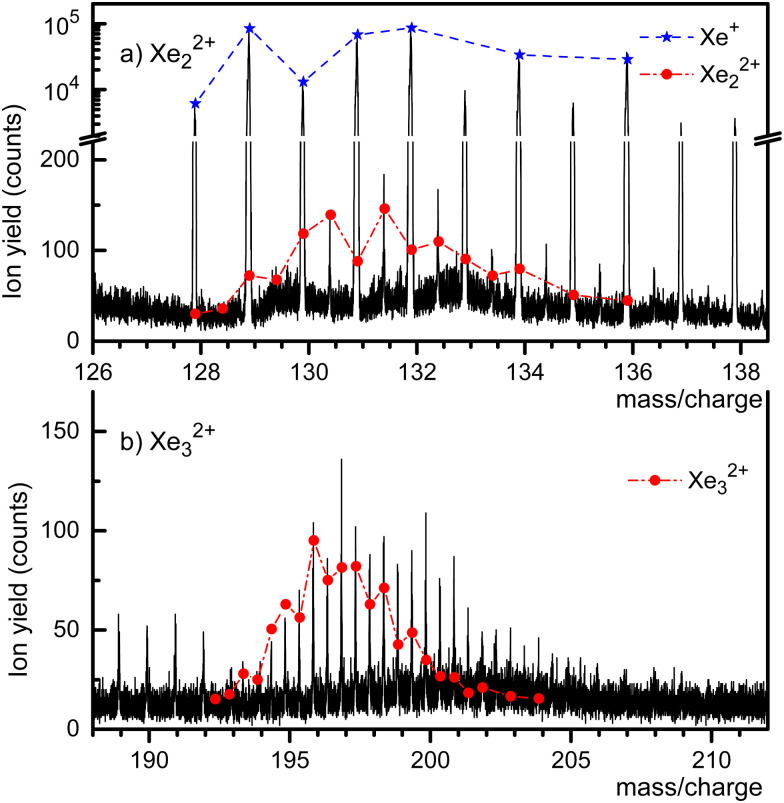
Sections of a mass spectrum of HNDs doped with Xe, with both ionizers turned on. Doubly charged dimers and trimers are observed in panels a and b, respectively. The expected abundance distributions are indicated by dash-dotted lines. Mass peaks at half-integer *m*/*z* values that are more intense than expected are due to He_*m*_Xe_*n*_^2+^ complexes.

A mass spectrum of HNDs doped with Xe, recorded with both ionizers turned on, is presented in Fig. 2. The isotopes of Xe, ^128^Xe through ^136^Xe, give rise to the prominent mass peaks in panel a (note the break in the *y*-scale). Several mass peaks appear at half-integer *m*/*z* values, starting at 130.5 which is mostly due to ^129^Xe^132^Xe^2+^ plus a small contribution from ^130^Xe^131^Xe^2+^. The expected abundance distribution, scaled to the value observed at *m*/*z* = 130.5, is shown by the dash-dotted line. The line underestimates the height of mass peaks further to the right, presumably because of contributions from He_*m*_Xe_2_^2+^. Likewise, the mass peaks in Fig. 2a at *m*/*z* = 133, 135, 137 and 138 are due to He_*m*_Xe^+^. It is impossible to set the pressure in the collision cell where He is stripped from the HNDs such that the embedded ions are completely stripped of the He without dissociating the bare dimers or trimers.

The half-integer mass peaks in Fig. 2b are due to the isotopologues of Xe_3_^2+^. The mismatch between the expected distribution of its isotopologues and the observed peak heights beyond *m*/*z* ≈ 196 indicates contributions from He_*m*_Xe_3_^2+^. On the other hand, there is no discernible contribution from He_*m*_Xe^+^ ions in this *m*/*z* range.

Now we turn to the computational investigation of the metastable ions. Somewhat simplified, the Coulomb barrier *E*_TS_ towards dissociation of doubly charged ions can be thought to arise due to the combination of a covalent bond potential with the Coulomb repulsion (Scheme 1; see ref. 9 for a detailed discussion of various contributing effects). The respective bound state is then often metastable towards dissociation, with the overall dissociation energy of *E*_dis_.

All investigated doubly-charged Ng_2_^2+^ ions, Ng = Ar, Kr, Xe, were found to be metastable with respect to dissociation into two Ng^+^ (^2^P) ions. Table 1 lists selected calculated properties of the ions at CCSD(T) and MRCI levels of theory; both methods predict the same optimal bond lengths within 0.012 Å. The intranuclear distance increases from about 2.02 Å for Ar_2_^2+^ through 2.32 Å for Kr_2_^2+^ to 2.69 Å for Xe_2_^2+^. A larger distance between nuclei leads to lower Coulomb repulsion as can be also seen from the dissociation energy to form Ng^+^ + Ng^+^ that is predicted as 4.75 eV (Ar_2_^2+^), 3.95 eV (Kr_2_^2+^) and 3.18 eV (Xe_2_^2+^).

**Table 1:** Calculated properties of Ng_2_^+^, Ng_2_^2+^, Ng_3_^+^, and Ng_3_^2+^ including dissociation of the doubly-charged species into Ng^+^ + Ng^+^ and Ng_2_^+^ + Ng^+^. See Scheme 1 for definition of the used nomenclature. The def2QZVPPD basis set was used for all calculations, see Methods for further details. Bond lengths in Ng_2_ are added for comparison

	Ar	Kr	Xe
*R*_e_ (Ng_2_)/Å, ref. 51	3.758	4.03	4.361
*R*_e_ (Ng_2_^+^)/Å, CCSD(T)	2.408	2.698	3.073
*R*_e_ (Ng_2_^2+^)/Å, CCSD(T)	2.026	2.324	2.688
*R*_e_ (Ng_2_^2+^)/Å, MRCI(10,11)	2.014	2.325	2.697
*R*_TS_ (Ng_2_^2+^)/Å, MRCI(10,11)	2.581	3.011	3.547
*E*_TS_ (Ng_2_^2+^)/eV, MRCI(10,11)	0.42	0.50	0.59
*E*_dis_ (Ng_2_^2+^)/eV, CCSD(T)	4.75	3.95	3.18
*R*_e_ (Ng_3_^+^)/Å, CCSD(T)	2.58	2.87	3.25
*R*_e_ (Ng_3_^2+^)/Å, CCSD(T)	2.35^a^	2.58	2.91
*R*_e_ (Ng_3_^2+^)/Å, MRCI(16,9)	—	2.65	2.97
*R*_e_ (Ng_3_^2+^)/Å, MRCI(16,10)	—	2.62	2.96
*R*_TS_ (Ng_3_^2+^)/Å, MRCI(16,9)	—	3.08; 2.93	3.74; 3.21
*R*_TS_ (Ng_3_^2+^)/Å, MRCI(16,10)	—	3.14; 2.84	3.78; 3.17
*E*_TS_ (Ng_3_^2+^)/eV, MRCI(16,9)	—	0.058	0.22
*E*_TS_ (Ng_3_^2+^)/eV, MRCI(16,10)	—	0.12	0.28
*E*_dis_ (Ng_3_^2+^)/eV, CCSD(T)	3.51	2.88	2.24

a A minimum is predicted with a barrier of 0.07 eV towards dissociation into Ar_2_^+^ and Ar^+^.

**Fig. 3:**
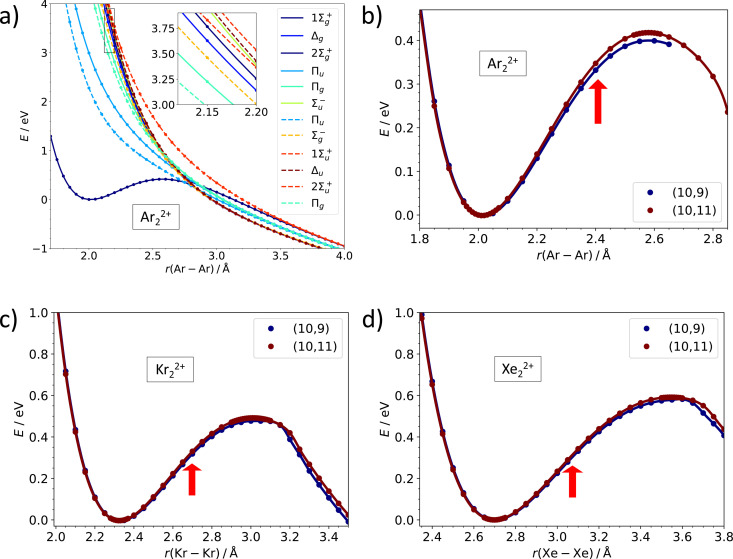
Multi-reference calculations on Ng_2_^2+^. (a) Analysis of electronic states along the dissociation coordinate in Ar_2_^2+^ as calculated at the MRCI(10,11)/def2QZVPPD level. Solid and dashed lines refer to singlet and triplet states, respectively. (b)–(d) Dissociation curves in Ar_2_^2+^, Kr_2_^2+^, and Xe_2_^2+^ as calculated at the MRCI/def2QZVPPD level with (10,9) and (10,11) active spaces. Red arrows indicate the equilibrium bond length in Ng_2_^+^. See the ESI,† for numerical values (Table S6, ESI†) and benchmarking calculations on Xe_2_^2+^ (Fig. S2, ESI†).

While the dissociation energy is relatively straightforward to calculate, the barrier against dissociation is harder to assess. The electronic structure of the Ar_2_^2+^ ion is analyzed in Fig. 3a. There are 36 electronic states corresponding to the asymptote of Ar^+^ (^2^P) + Ar^+^ (^2^P). Among these, only one ^1^Σ^+^_g_ molecular term is bound at a short internuclear distance while all other terms (^1^Σ^+^_g_, ^1^Σ^−^_u_, ^1^Π_g_, ^1^Π_u_, ^1^Δ_g_, ^3^Σ^−^_g_, 2x ^3^Σ^+^_u_, ^3^Π_g_, ^3^Π_u_, ^3^Δ_u_) correspond to purely dissociative states, analogously to the situation in the isoelectronic Cl_2_ system.^43^ The potential energy curves in Fig. 3a illustrate the inherent problem in modeling the Coulomb barrier in Ng_2_^2+^ ions. In the vicinity of the transition state that arises as a combination of the covalent bond potential and the Coulomb interaction, the curve of the ^1^Σ^+^_g_ term is crossed by the dissociative states. Single-reference methods might struggle to describe this region and, therefore, multi-reference approach was used for calculating the Coulomb barriers.

Our MRCI calculations predict almost the same barrier against dissociation of 0.4–0.6 eV for all three Ng_2_^2+^ ions, the bond length in the transition state is by about 30% longer than in the metastable minimum. In the dissociation curves in Fig. 3b–d, the turn in the curve between the regions of covalent bonding and Coulomb dissociation is clearly visible; the active spaces include all orbitals correlating to p orbitals of Ng^+^ and seem to be converged with respect to the number of virtual orbitals (see also the ESI†).

**Fig. 4:**
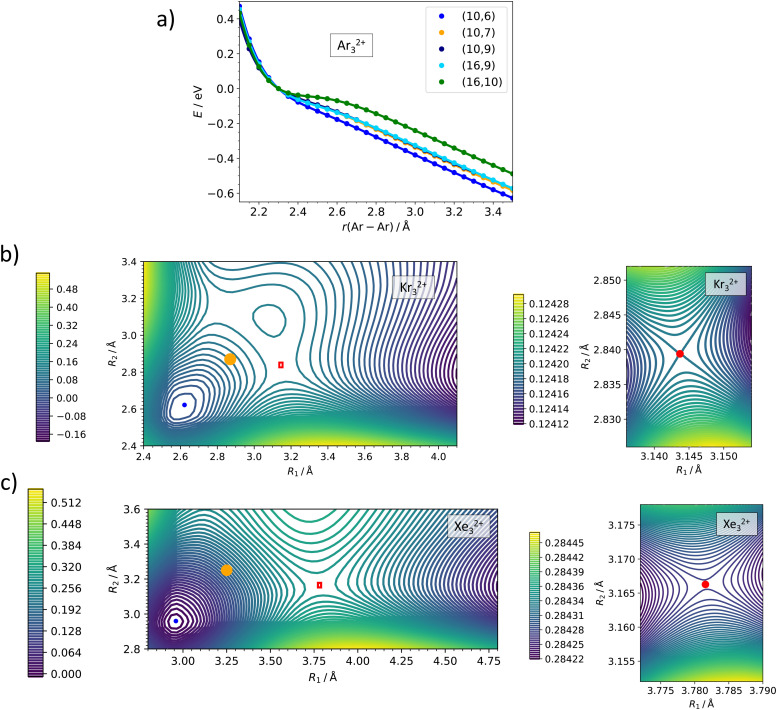
Multi-reference calculations on Ng_3_^2+^. (a) Analysis of instability of Ar_3_^2+^ as calculated at the MRCI/def2QZVPPD level with various active spaces. (b) and (c) Two-dimensional contour plots for dissociation of Kr_3_^2+^ and Xe_3_^2+^ as calculated at the MRCI(16,10)/def2QZVPPD level. Orange points show the optimal bond lengths in Ng_3_^+^, red rectangles show the region of the zoomed plot, red points the position of the transition state. See the ESI,† for benchmarking calculations on Xe_3_^2+^ (Fig. S3, ESI†).

In doubly-charged trimers, Ng_3_^2+^, the minimum is predicted to be a linear structure of *D*_∞h_ symmetry, in agreement with a previous study on He_3_^2+^.^44^ While Kr_3_^2+^ and Xe_3_^2+^ ions are predicted to be metastable towards dissociation into Ng^+^ + Ng_2_^+^, our calculations are not unequivocal with respect to stability of Ar_3_^2+^. At the CCSD(T) level, a minimum is predicted with the bond length of 2.35 Å (Table 1), with a barrier towards dissociation of 0.07 eV, above the calculated zero-point energy of the symmetric stretch mode of 0.014 eV. At the MRCI level, none of the investigated active spaces predicts a minimum along the Ar–Ar–Ar dissociation coordinate (Fig. 4a). The *a posteriori* Davidson correction (MRCI + Q) predicts a minimum with a barrier against symmetric dissociation of about 0.05 eV (Fig. S5, ESI†). While the MRCI approach might be still considerably influenced by the active space size and the absence of proper treatment of triples and higher excitations, the CCSD(T) method might suffer from an inappropriate Hartree–Fock wave function used as the basis of the calculation; it is therefore complicated to give a definitive verdict on Ar_3_^2+^ (in)stability.

In Kr_3_^2+^ and Xe_3_^2+^, optimal bond lengths at MRCI and CCSD(T) levels differ by about 0.1 Å, *i.e.*, considerably more than in Ng_2_^2+^ systems, hinting towards a more involved electronic structure. The transition state for dissociation to Ng_2_^+^ + Ng^+^ is asymmetric (Fig. 4b and c), reflecting the formation of the Ng_2_^+^ ion along the reaction pathway. Dissociation takes place over a small barrier of about 0.1 eV and 0.3 eV for Kr_3_^2+^ and Xe_3_^2+^, respectively (Table 1). However, the precise value of the barrier is affected considerably by parametrization of the MRCI method; from our benchmarks we suggest an error of the barrier energy of around 0.1 eV for Xe_3_^2+^ arising from the active space size (see Fig. S3, ESI†). All MRCI calculations consistently predict a metastable bound structure for Kr_3_^2+^ and Xe_3_^2+^.

## 4 Discussion

Isotopologues of dicationic dimers and trimers of Kr and Xe whose mass/charge ratio is half integer have been identified in high-resolution mass spectra. Their appearance implies that they have survived intact from the time of ion extraction until the reflectron in the TOF-MS, which measures about 200–300 μs.

For Ar, Kr, and Xe, the calculated internuclear separation *R*_TS_ in the transition state of Ng_2_^2+^ (^1^Σ^+^_g_) exceeds the separation in the minimum of the ground state of Ng_2_^+^ (^2^Σ^+^_u_), see Table 1 and Fig. 3. Hence, a vertical transition from Ng_2_^+^ (^2^Σ^+^_u_) into Ng_2_^2+^ (^1^Σ^+^_g_) will likely end up to the left of the transition state in the PEC, resulting in a vibrationally excited quasi-bound dication. We do not know what the lifetime of these species with respect to tunneling would be in vacuum, but their interaction with the HND offers a pathway for vibrational relaxation and stabilization. While HNDs often fail to suppress ionization-induced fragmentation,^45–47^ there are notable exceptions.^48,49^ An ion formed on an attractive part of the PEC has more time for vibrational relaxation than one that is formed on a purely repulsive part.

Our mass spectra of Kr and Xe-doped HNDs reveal the presence of dicationic trimers which have so far not been observed for any noble gas. There are several factors that favor their formation from the monocations as opposed to from the neutral. First, the dications are linear symmetric molecules like their singly charged counterparts.^50^ Second, the bond length in the dication is not much shorter than in monocation (2.91 Å *versus* 3.25 Å for Xe); again, when Ng_3_^+^ is ionized to form Ng_3_^2+^, it would reach the part of the potential energy surface from which it can reach the metastable minimum (Fig. 4b and c). In contrast, the neutral trimer would have an equilateral geometry with a bond length of about 4.36 Å;^51^ the Franck–Condon factor for a transition into the region of the metastable potential well of Ng_3_^2+^ would be negligible.

We do not observe dicationic tetramers, nor slightly larger dications. An *ab initio* study of dicationic helium clusters has suggested quasistable He_*n*_^2+^ up to *n* = 6, with a covalently bound trimer or tetramer core.^44^ We succeeded to optimize a Xe_4_^2+^ ion of a linear structure at the CCSD/def2TZVP level, with bond lengths of 3.11, 3.02, and 3.11 Å. The question is, though, how fast charge transfer would lead to two separate charge centers within the complex, which would then quickly undergo fission into two singly charged clusters unless they contain dozens of atoms.^52,53^ For Xe, for example, dicationic clusters containing at least 47 atoms have been observed upon electron ionization of neutral clusters.^54^ With the current experimental approach we observe Xe_*n*_^2+^ containing as few as 25 atoms if the HNDs are heavily doped, but no dications appear between *n* = 3 and 25. These findings will be reported in a future paper.

We have no direct information about the processes that occur in the doped HNDs when passing through the second ionizer or the collision cell where excess helium is being removed. We do not know for sure what the singly charged precursors of the observed dicationic dimers and trimers are. Ionization-induced fragmentation is a ubiquitous phenomenon in cluster science. It is conceivable that Ng atoms were lost upon formation of a long-lived dication Ng_*n*_^2+^, *n* = 2, 3.

Another issue is the mechanism by which dications are formed in the second ionization region. Broadly speaking, there are three pathways by which an incident electron may ionize a doped HND. First, the electron may directly ionize the dopant. Second, the incident electron may form He^+^ which moves toward the dopant by resonant charge hopping and ionizes the dopant by charge transfer (or the charge may be trapped on He_2_^+^ which then migrates towards the dopant). Third, the incident electron may electronically excite the helium, and the dopant is ionized by an intracluster Penning mechanism.

The efficiency of process (1) is small compared to (2) and (3) unless the droplet is very small. Process (2) dominates if the dopant is heliophilic, but it can be excluded in the situation considered here because the charged dopant will repel He^+^. Penning ionization (process 3) *via* formation of He* or 
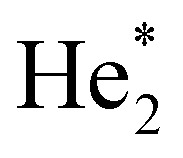
 dominates if the dopant is heliophobic and resides on the surface.^35,37–39^ In a multiply charged droplet, the dopant ions reside close to the surface because of Coulomb repulsion, but they will be surrounded by one or a few solvation layers.^36^ Hence, it is not clear if heliophobic He* or 
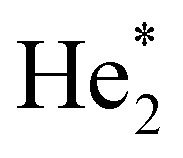
 will be able to Penning ionize the dopant ions. However, photoelectron experiments of HNDs doped with noble gas atoms (which are heliophilic) have shown that Penning ionization does occur in small HNDs if the incident radiation leads to excitation of the 1s2p or higher bands.^55–57^ A better understanding of the ionization mechanism could be obtained if the ion yield were to be measured *versus* the energy of the incident electrons, but this kind of experiment would require higher ion yields than attainable right now.

Finally we discuss the non-observation of doubly charged argon complexes which may be due to technical reasons. First, extraction of ions that exit from the He collision cell is disfavored for light ions with *m*/*z* well below 100. Second, the isotope pattern of argon disfavors mass spectrometric detection of dications. The Ar isotopes and their abundances are ^36^Ar (0.34%), ^38^Ar (0.06%), ^40^Ar (99.90%). Thus, neither Ar_2_^2+^ nor Ar_3_^2+^ have any isotopologues with a half-integer *m*/*z* value. In summary, the absence of doubly charged Ar dimers or trimers in the current work conveys no definitive information about their stability or lack thereof.

## 5 Conclusions

We report the observation of Kr_2_^2+^, Kr_3_^2+^, Xe_2_^2+^, and Xe_3_^2+^ in helium nanodroplets *via* post-ionization of singly-charged ions. Computational investigations suggest that when Ng_2_^+^ or Ng_3_^+^ is ionized, one reaches the potential energy surface in the region where doubly-charged ions are metastable, and dissociation is prevented by a small Coulomb barrier. Notably, the Coulomb barrier in Kr_3_^2+^ is predicted to be around 0.1 eV, the ions are thus very efficiently stabilized by helium evaporation. We observed neither Ar_2_^2+^ nor Ar_3_^2+^, although our *ab initio* calculations predict Ar_2_^2+^ to be metastable with a considerable Coulomb barrier of 0.4 eV, preventing dissociation into Ar^+^ + Ar^+^. Most importantly, we have shown that our method of sequential double ionization can successfully prepare metastable, multiply-charged ions that would be hard or even impossible to form in other experimental approaches.

## Data availability

The data that support the findings of this study are available from the corresponding authors upon reasonable request.

## Author contributions

Conceptualization, E. G. (E. Gruber) and P. S. (P. Scheier); formal analysis, I. S. (I. Stromberg), S. B. (S. Bergmeister), M. M. D. (M. Mahmoodi-Darian), and E. G.; funding acquisition, M. O. (M. Ončák) and P. S.; investigation, I. S., S. B., E. G., G. S. (G. Schöpfer) and M. O.; methodology, E. G., M. O. and P. S.; project administration, M. O. and P. S.; resources, M. O. and P. S.; software, G. S. and M. O.; supervision, E. G., P. S. and M. O.; visualization, I. S., E. G., M. O. and O. E.; writing—original draft, M. O. and O. E.; writing—review & editing, I. S., S. B., E. G., M. M. D., G. S., M. O., P. S. and O. E. All authors have read and agreed to the published version of the manuscript.

## Conflicts of interest

We declare no conflict of interest.

## Supplementary Material

CP-026-D4CP00465E-s001

## References

[cit1] Pauling L. (1933). The Normal State of the Helium Molecule-Ions He^2+^ and He^2++^. J. Chem. Phys..

[cit2] Guilhaus M., Brenton A. G., Beynon J. H., Rabrenovic M., von Rague Schleyer P. (1984). First observation of He_2_^2+^: charge stripping of He^2+^ using a double-focusing mass spectrometer. J. Phys. B.

[cit3] Guilhaus M., Brenton A. G., Beynon J. H., Rabrenović M., von Ragué Schleyer P. (1985). He_2_^2+^, the experimental detection of a remarkable molecule. J. Chem. Soc., Chem. Commun..

[cit4] Belkacem A., Kanter E. P., Mitchell R. E., Vager Z., Zabransky B. J. (1989). Measurement of the ultrashort bond length in He^2++^. Phys. Rev. Lett..

[cit5] Ben-Itzhak I., Gertner I., Bortman D., Zajfman D. (1990). Formation of doubly charged molecular ion Ne_2_^2+^ in sub-MeV stripping collision Ne^2+^ + Ar → Ne_2_^2+^. Phys. Rev. A: At., Mol., Opt. Phys..

[cit6] Tessner T., Drachsel W., Dirks J., Block J. H. (1994). Observation of doubly charged Ne_2_ molecular ions by field desorption. Int. J. Mass Spectrom. Ion Processes.

[cit7] Ackermann J., Hogreve H. (1992). On the metastability of the ^1^Σ^+^_g_ ground state of He_2_^2+^ and Ne_2_^2+^: a case study of binding metamorphosis. J. Phys. B.

[cit8] Ackermann J., Hogreve H. (2017). Stability and spectral properties of the dication Ne_2_^2+^. Phys. Chem. Chem. Phys..

[cit9] Fantuzzi F., Cardozo T. M., Nascimento M. A. C. (2017). On the metastability of doubly charged homonuclear diatomics. Phys. Chem. Chem. Phys..

[cit10] Grandinetti F. (2011). Gas-Phase Ion Chemistry of the Noble Gases: Recent Advances and Future Perspectives. Eur. J. Mass Spectrom..

[cit11] Jahnke T., Hergenhahn U., Winter B., Dörner R., Frühling U., Demekhin P. V., Gokhberg K., Cederbaum L. S., Ehresmann A., Knie A., Dreuw A. (2020). Interatomic and Intermolecular Coulombic Decay. Chem. Rev..

[cit12] Saito N., Morishita Y., Suzuki I. H., Stoychev S. D., Kuleff A. I., Cederbaum L. S., Liu X.-J., Fukuzawa H., Prümper G., Ueda K. (2007). Evidence of radiative charge transfer in argon dimers. Chem. Phys. Lett..

[cit13] Lezius M., Dobosz S., Normand D., Schmidt M. (1998). Explosion Dynamics of Rare Gas Clusters in Strong Laser Fields. Phys. Rev. Lett..

[cit14] Manschwetus B., Rottke H., Steinmeyer G., Foucar L., Czasch A., Schmidt-Böcking H., Sandner W. (2010). Mechanisms underlying strong-field double ionization of argon dimers. Phys. Rev. A: At., Mol., Opt. Phys..

[cit15] Matsumoto J., Leredde A., Flechard X., Hayakawa K., Shiromaru H., Rangama J., Zhou C. L., Guillous S., Hennecart D., Muranaka T., Mery A., Gervais B., Cassimi A. (2010). Asymmetry in Multiple-Electron Capture Revealed by Radiative Charge Transfer in Ar Dimers. Phys. Rev. Lett..

[cit16] Morishita Y., Liu X.-J., Saito N., Lischke T., Kato M., Prümper G., Oura M., Yamaoka H., Tamenori Y., Suzuki I. H., Ueda K. (2006). Experimental Evidence of
Interatomic Coulombic Decay from the Auger Final States in Argon Dimers. Phys. Rev. Lett..

[cit17] Ren X., Jabbour Al Maalouf E., Dorn A., Denifl S. (2016). Direct evidence of two interatomic relaxation mechanisms in argon dimers ionized by electron impact. Nat. Commun..

[cit18] Rühl E. (2003). Core level excitation, ionization, relaxation, and fragmentation of free clusters. Int. J. Mass Spectrom..

[cit19] Wu J., Vredenborg A., Ulrich B., Schmidt L. P. H., Meckel M., Voss S., Sann H., Kim H., Jahnke T., Dörner R. (2011). Multiple Recapture of Electrons in Multiple Ionization of the Argon Dimer by a Strong Laser Field. Phys. Rev. Lett..

[cit20] Zhu X. L., Yan S., Feng W. T., Ma X., Chuai X. Y., Guo D. L., Gao Y., Zhang R. T., Zhang P., Zhang S. F., Zhao D. M., Xu S., Wang H. B., Huang Z. K., Qian D. B. (2018). Orientation effect in Ar dimer fragmentation by highly charged ion impact. J. Phys. B.

[cit21] Ackermann J., Hogreve H. (1993). A CI study of quasi-bound Ar_2_^2+^. Chem. Phys. Lett..

[cit22] Cachoncinlle C., Pouvesle J. M., Durand G., Spiegelmann F. (1992). Theoretical study of the electronic structure of Ar^2++^. J. Chem. Phys..

[cit23] Lablanquie P., Aoto T., Hikosaka Y., Morioka Y., Penent F., Ito K. (2007). Appearance of interatomic Coulombic decay in Ar, Kr, and Xe homonuclear dimers. J. Chem. Phys..

[cit24] Hoener M., Bostedt C., Schorb S., Thomas H., Foucar L., Jagutzki O., Schmidt-Böcking H., Dörner R., Möller T. (2008). From fission to explosion: momentum-resolved survey over the Rayleigh instability barrier. Phys. Rev. A: At., Mol., Opt. Phys..

[cit25] Mathur D., Rajgara F. A. (2010). Communication: Ionization and Coulomb explosion of Xenon clusters by intense, few-cycle laser pulses. J. Chem. Phys..

[cit26] Murakami H., Iwayama H., Nagaya K., Yao M. (2008). Fragmentation channels of K-shell excited rare-gas clusters studied by multiple-ion coincidence momentum imaging. J. Chem. Phys..

[cit27] Treshchalov A. B., Lissovski A. A. (2012). Multi-band spectral structure and kinetics of the third continua in Ar, Kr and Xe gases excited by a pulsed discharge. Eur. Phys. J. D.

[cit28] Albertini S., Bergmeister S., Laimer F., Martini P., Gruber E., Zappa F., Ončák M., Scheier P., Echt O. (2021). SF_6_^+^: Stabilizing Transient Ions in Helium Nanodroplets. J. Phys. Chem. Lett..

[cit29] Gruber E., Kollotzek S., Bergmeister S., Zappa F., Ončák M., Scheier P., Echt O. (2022). Phenanthrene: establishing lower and upper bounds to the binding energy of a very weakly bound anion. Phys. Chem. Chem. Phys..

[cit30] Kollotzek S., Izadi F., Meyer M., Bergmeister S., Zappa F., Denifl S., Echt O., Scheier P., Gruber E. (2022). Stabilization of phenanthrene anions in helium nanodroplets. Phys. Chem. Chem. Phys..

[cit31] Ganner L. , Bergmeister S., Lorenz L., Ončák M., Scheier P. and Gruber E., Charging up the cold: formation of doubly- and triply-charged fullerene dimers in superfluid helium nanodroplets, *arXiv*, 2024, preprint, arXiv:2312.05151 10.48550/arXiv.2312.05151 39073966

[cit32] Bergmeister S., Ganner L., Locher J., Zappa F., Scheier P., Gruber E. (2023). Spectroscopy of helium-tagged molecular ions—Development of a novel experimental setup. Rev. Sci. Instrum..

[cit33] Laimer F., Kranabetter L., Tiefenthaler L., Albertini S., Zappa F., Ellis A. M., Gatchell M., Scheier P. (2019). Highly Charged Droplets of Superfluid Helium. Phys. Rev. Lett..

[cit34] Mateo D., Eloranta J. (2014). Solvation of Intrinsic Positive Charge in Superfluid Helium. J. Phys. Chem. A.

[cit35] Mauracher A., Echt O., Ellis A. M., Yang S., Bohme D. K., Postler J., Kaiser A., Denifl S., Scheier P. (2018). Cold physics and chemistry: collisions, ionization and reactions inside helium nanodroplets close to zero K. Phys. Rep..

[cit36] Feinberg A. J., Laimer F., Tanyag R. M. P., Senfftleben B., Ovcharenko Y., Dold S., Gatchell M., O’Connell-Lopez S. M. O., Erukala S., Saladrigas C. A., Toulson B. W., Hoffmann A., Kamerin B., Boll R., Fanis A., de, Grychtol P., Mazza T., Montano J., Setoodehnia K., Lomidze D., Hartmann R., Schmidt P., Ulmer A., Colombo A., Meyer M., Möller T., Rupp D., Gessner O., Scheier P., Vilesov A. F. (2022). X-ray diffractive imaging of highly ionized helium nanodroplets. Phys. Rev. Res..

[cit37] Ziemkiewicz M. P., Neumark D. M., Gessner O. (2015). Ultrafast electronic dynamics in helium nanodroplets. Int. Rev. Phys. Chem..

[cit38] Scheidemann A. A., Kresin V. V., Hess H. (1997). Capture of lithium by ^4^He clusters: surface adsorption, Penning ionization, and formation of HeLi^+^. J. Chem. Phys..

[cit39] Schöbel H., Bartl P., Leidlmair C., Daxner M., Zöttl S., Denifl S., Märk T. D., Scheier P., Spångberg D., Mauracher A., Bohme D. K. (2010). Sequential Penning Ionization: Harvesting Energy with Ions. Phys. Rev. Lett..

[cit40] Frisch M. J. , Trucks G. W., Schlegel H. B., Scuseria G. E., Robb M. A., Cheeseman J. R., Scalmani G., Barone V., Petersson G. A., Nakatsuji H., Li X., Caricato M., Marenich A. V., Bloino J., Janesko B. G., Gomperts R., Mennucci B., Hratchian H. P., Ortiz J. V., Izmaylov A. F., Sonnenberg J. L., Williams-Young D., Ding F., Lipparini F., Egidi F., Goings J., Peng B., Petrone A., Henderson T., Ranasinghe D., Zakrzewski V. G., Gao J., Rega N., Zheng G., Liang W., Hada M., Ehara M., Toyota K., Fukuda R., Hasegawa J., Ishida M., Nakajima T., Honda Y., Kitao O., Nakai H., Vreven T., Throssell K., Montgomery Jr. J. A., Peralta J. E., Ogliaro F., Bearpark M. J., Heyd J. J., Brothers E. N., Kudin K. N., Staroverov V. N., Keith T. A., Kobayashi R., Normand J., Raghavachari K., Rendell A. P., Burant J. C., Iyengar S. S., Tomasi J., Cossi M., Millam J. M., Klene M., Adamo C., Cammi R., Ochterski J. W., Martin R. L., Morokuma K., Farkas O., Foresman J. B. and Fox D. J., Gaussian 16 Rev. A.03, Gaussian Inc., Wallingford, CT, 2016

[cit41] Werner H.-J., Knowles P. J., Knizia G., Manby F. R., Schütz M. (2012). Molpro: a general-purpose quantum chemistry program package. Wiley Interdiscip. Rev.: Comput. Mol. Sci..

[cit42] Werner H.-J. , Knowles P. J., Lindh R., Manby F. R., Schütz M., Celani P., Korona T., Mitrushenkov A., Rauhut G., Adler T. B., Amos R. D., Bernhardsson A., Berning A., Cooper D. L., Deegan M. J. O., Dobbyn A. J., Eckert F., Goll E., Hampel C., Hetzer G., Hrenar T., Knizia G., Köppl C., Liu Y., Lloyd A. W., Mata R. A., May A. J., McNicholas S. J., Meyer W., Mura M. E., Nicklaß A., Palmieri P., Pflüger K., Pitzer R., Reiher M., Schumann U., Stoll H., Stone A. J., Tarroni R., Thorsteinsson T., Wang M. and Wolf A., MOLPRO, version 2012.1, a Package of Ab Initio Programs, Stuttgart, 2012, see https://www.molpro.net

[cit43] Johnsen A. J., Alekseyev A. B., Balint-Kurti G. G., Brouard M., Brown A., Buenker R. J., Campbell E. K., Kokh D. B. (2012). A complete quantum mechanical study of chlorine photodissociation. J. Chem. Phys..

[cit44] Hogreve H. (1992). On the resonance structure of the adiabatic hypersurfaces of small doubly charged helium clusters He_*n*_^++^, *n* = 3–6. J. Chem. Phys..

[cit45] Mudrich M., Stienkemeier F. (2014). Photoionisaton of pure and doped helium nanodroplets. Int. Rev. Phys. Chem..

[cit46] Schöbel H., Bartl P., Leidlmair C., Denifl S., Echt O., Märk T. D., Scheier P. (2011). High-resolution mass spectrometric study of pure helium droplets, and droplets doped with krypton. Eur. Phys. J. D.

[cit47] Yang S., Brereton S. M., Wheeler M. D., Ellis A. M. (2005). Soft or hard ionization of molecules in helium nanodroplets? An electron impact investigation of alcohols and ethers. Phys. Chem. Chem. Phys..

[cit48] Ellis A. M., Yang S. F. (2015). Role of Helium Droplets in Mass Spectra of Diatomics: Suppression of Dissociative Reactions. Chin. J. Chem. Phys..

[cit49] Ren Y., Kresin V. V. (2008). Suppressing the fragmentation of fragile molecules in helium nanodroplets by coembedding with water: possible role of the electric dipole moment. J. Chem. Phys..

[cit50] Daskalopoulou M., Böhmer H.-U., Peyerimhoff S. D. (1990). Multi-reference configuration interaction calculations on the systems Xe_2_^+^ and Xe_3_^+^. Z. Phys. D: At., Mol. Clusters.

[cit51] Linstrom P. J., Mallard W. G. (2001). The NIST Chemistry WebBook: A Chemical Data Resource on the Internet. J. Chem. Eng. Data.

[cit52] Echt O., Kreisle D., Recknagel E., Saenz J. J., Casero R., Soler J. M. (1988). Dissociation channels of multiply charged van der Waals clusters. Phys. Rev. A: At., Mol., Opt. Phys..

[cit53] Echt O., Scheier P., Märk T. D. (2002). Multiply charged clusters. C. R. Phys..

[cit54] Scheier P., Märk T. D. (1987). Doubly charged argon clusters and their critical size. J. Chem. Phys..

[cit55] Wang C. C., Kornilov O., Gessner O., Kim J. H., Peterka D. S., Neumark D. M. (2008). Photoelectron imaging of helium droplets doped with Xe and Kr atoms. J. Phys. Chem. A.

[cit56] Buchta D., Krishnan S. R., Brauer N. B., Drabbels M., O'Keeffe P., Devetta M., Di Fraia M., Callegari C., Richter R., Coreno M., Prince K. C., Stienkemeier F., Moshammer R., Mudrich M. (2013). Charge Transfer and Penning Ionization of Dopants in or on Helium Nanodroplets Exposed to EUV Radiation. J. Phys. Chem. A.

[cit57] Latief L. B., Shcherbinin M., Krishnan S. R., Richter R., Pfeifer T., Mudrich M. (2020). Direct inner-shell photoionization of Xe atoms embedded in helium nanodroplets. J. Phys. B.

