# Supplementary material for: Doubly charged dimers and trimers of heavy noble gases
Source: Phys Chem Chem Phys. 2024 Mar 23;26(15):11482–90. doi: 10.1039/d4cp00465e (PMC11022278; doi:10.1039/d4cp00465e)
Supplement: CP-026-D4CP00465E-s001 [file CP-026-D4CP00465E-s001.pdf]

Electronic Supplementary Material (ESI) for Physical Chemistry Chemical Physics.
This journal is © the Owner Societies 2024

Electronic supplementary information

Doubly Charged Dimers and Trimers of Heavy Noble Gases

Gabriel Schöpfer,[1a] Stefan Bergmeister,[1a] Milan Ončák,[1*] Ianessa Stromberg,[1,2] Masoomeh Mahmoodi-Darian,[1] Paul Scheier,[1] Olof Echt[1,3*], Elisabeth Gruber[1]

1 Institut für Ionenphysik und Angewandte Physik, Universität Innsbruck, Innsbruck, Austria

2 School of Chemistry, University of Edinburgh, Edinburgh, United Kingdom

3 Department of Physics, University of New Hampshire, Durham, USA

a The authors contributed equally

ORCID and Email:

G. Schöpfer	0000-0001-5657-2671	Gabriel.Schoepfer@uibk.ac.at
S. Bergmeister	0000-0003-0848-3038	Stefan.Bergmeister@uibk.ac.at
M. Ončák	0000-0002-4801-3068	Milan.Oncak@uibk.ac.at
I. Stromberg	0009-0000-4524-4736	Ianessastromberg@gmail.com
M. Mahmoodi-Darian	0000-0003-4350-4770	Masoomeh.Mahmoodi@uibk.ac.at
P. Scheier	0000-0002-7480-6205	Paul.Scheier@uibk.ac.at
O. Echt	0000-0002-0970-1191	Olof.Echt@unh.edu
E. Gruber	0000-0002-1195-3638	E.Gruber@uibk.ac.at

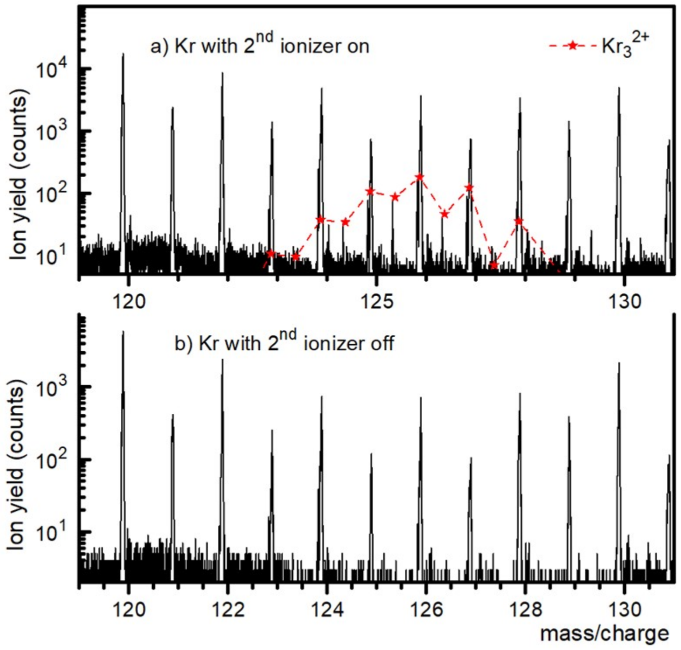

Fig. S1
A mass spectrum of HNDs doped with Kr. Isotopologues of Kr_3^{2+} appear in Fig. 1a when both ionizers are turned on; the expected abundance distribution of their isotopologues is indicated by the dash-dotted line. No Kr_3^{2+} appears when the 2nd ionizer is turned off (Fig. 1b).

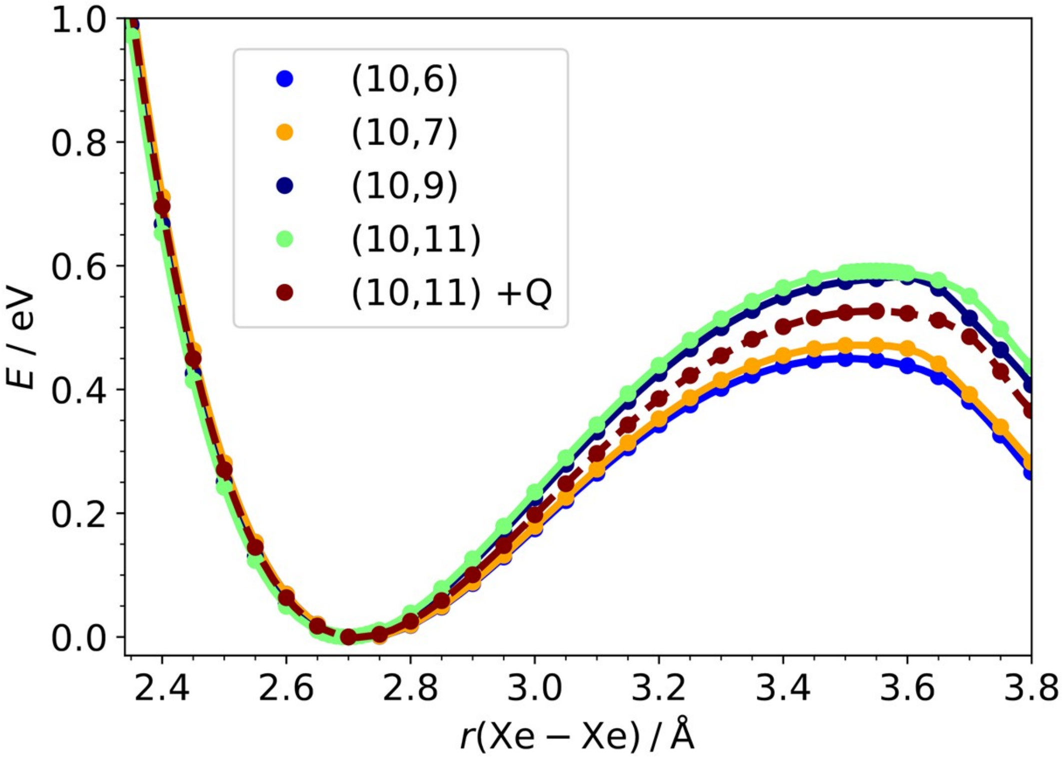

Fig. S2
Benchmarking of active space for Xe_2^{2+} at the MRCI/def2QZVPPD level. The dashed line refers to a calculation including relaxed Davidson correction (MRCI+Q).

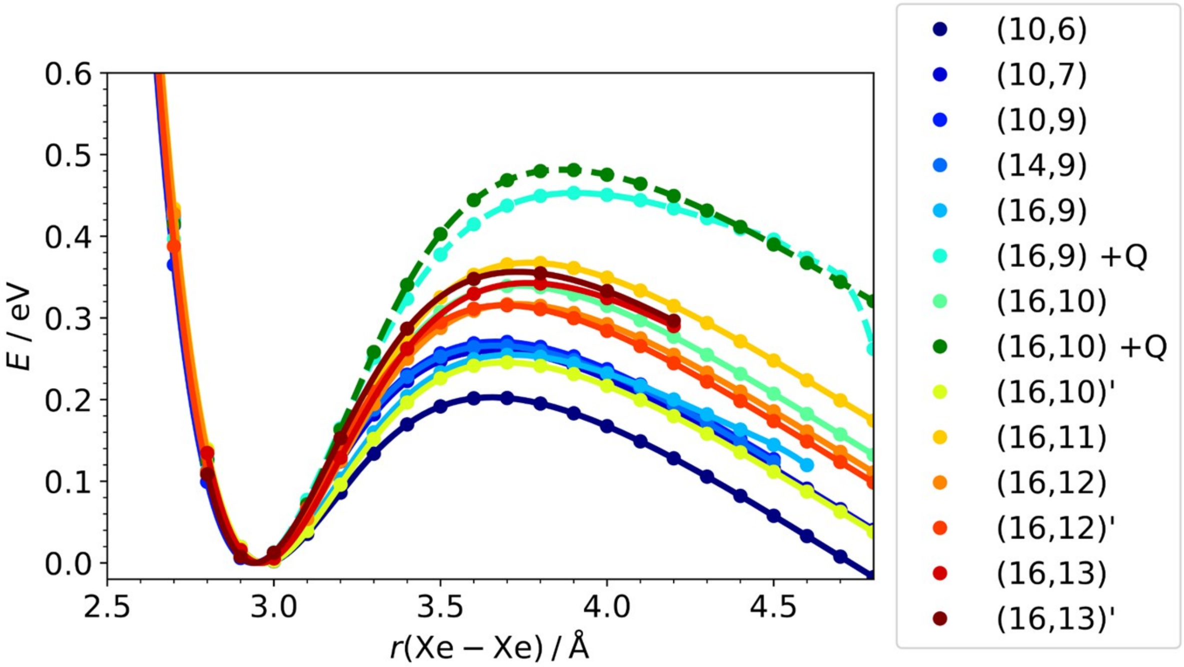

Fig. S3
Benchmarking of active space for Xe_3^{2+} at the MRCI/def2QZVPPD level. Dashed lines refer to calculations including relaxed Davidson correction (MRCI+Q).

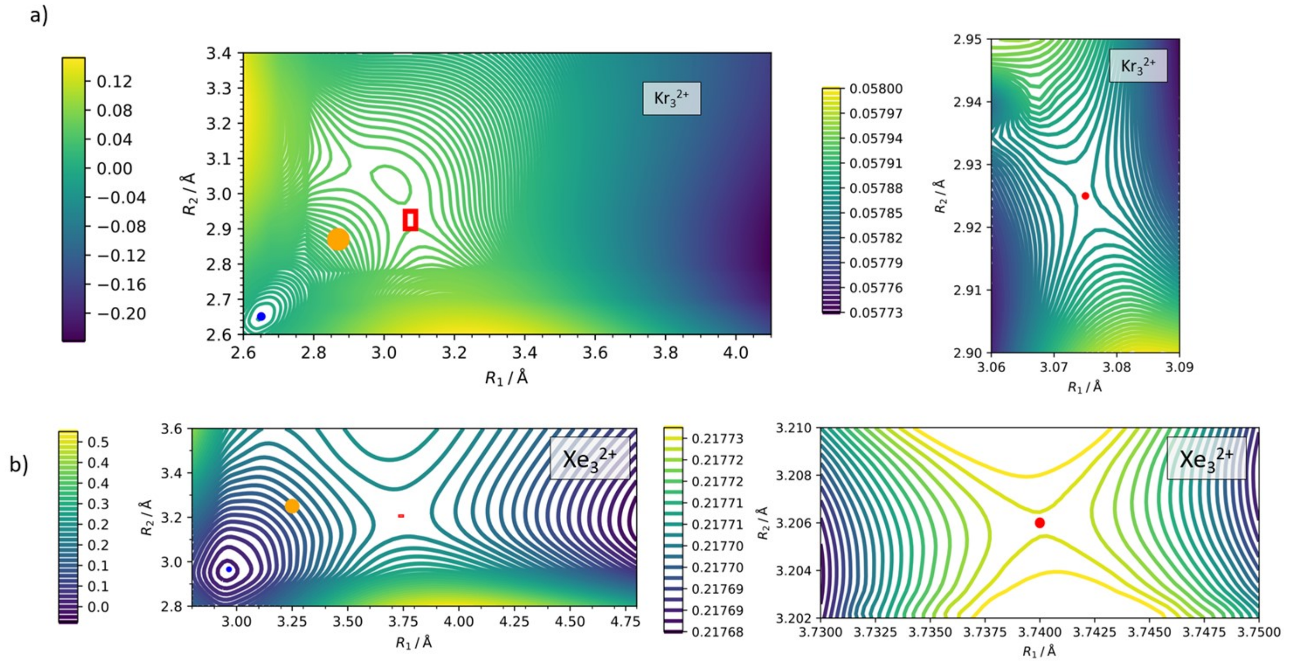

Fig. S4
Two-dimensional contour plots for dissociation in Kr_3^{2+} and Xe_3^{2+} as calculated at the MRCI(16,9)/def2QZVPPD level. Orange points show the optimal bond lengths in Ng_3^+ at the CCSD(T)/def2QZVPPD level, red rectangles show the region of the zoomed plot on the right and red points the position of the transition state for dissociation into Ng_2^+ and Ng^+.

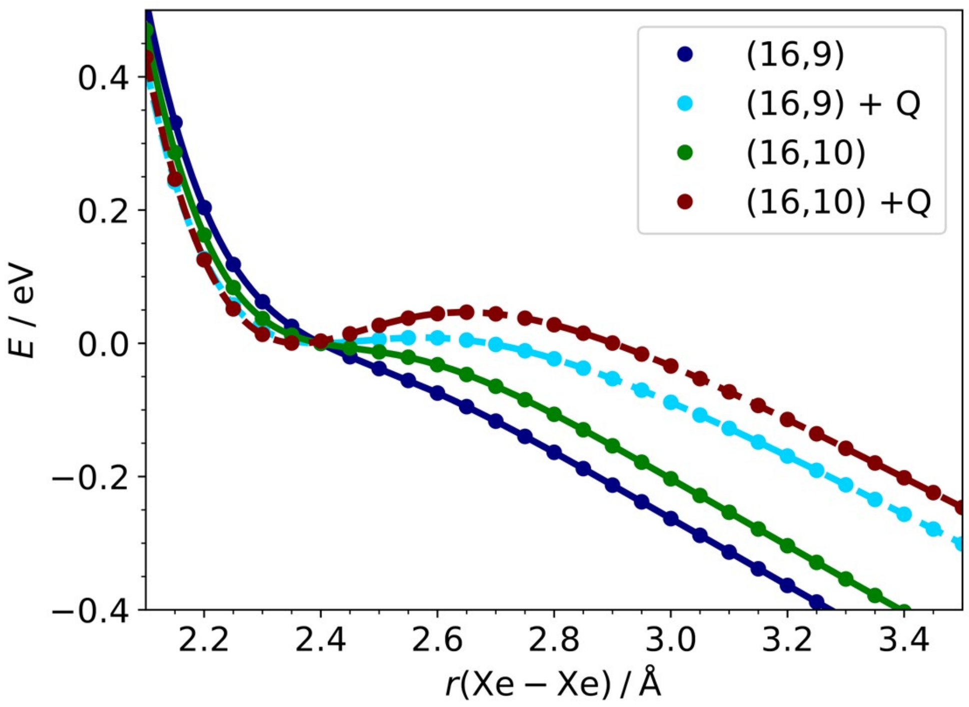

Fig. S5
Benchmarking for Ar_3^{2+} at the MRCI/def2QZVPPD level. Dashed lines refer to calculations including relaxed Davidson correction (MRCI+Q).

Tab. S1
Definition of active spaces for Ng_2^{2+} and Ng_3^{2+}, Ng = Ar, Kr, Xe in the $D_{\infty h}$ point group used for 1D scans (in Ng_3^{2+}, both bond lengths were scanned simultaneously).

**Ar_2^{2+} (34 electrons, $D_{\infty h}$)**	σ_g^+	σ_g^-	π_g	δ_g	σ_u^+	σ_u^-	π_u	δ_u
closed orbitals	1,2,3,4		1		1,2,3,4		1	
active in (10,9)	5,6		2		5		2,3	
active in (10,11)	5,6		2	1	5		2,3	

**Kr_2^{2+} (70 electrons, $D_{\infty h}$)**	σ_g^+	σ_g^-	π_g	δ_g	σ_u^+	σ_u^-	π_u	δ_u
closed orbitals	1,2,3,4,5,6,7		1,2,3	1	1,2,3,4,5,6,7		1,2,3	1
active in (10,9)	8,9		4		8		4,5	
active in (10,11)	8,9		4	2	8		4,5	

**Xe_2^{2+} (50 electrons, $D_{\infty h}$)**	σ_g^+	σ_g^-	π_g	δ_g	σ_u^+	σ_u^-	π_u	δ_u
closed orbitals	1,2,3,4		1,2	1	1,2,3,4		1,2	1
active in (10,6)	5		3		5		3	
active in (10,7)	5,6		3		5		3	
active in (10,9)	5,6		3		5		3,4	
active in (10,11)	5,6		3	2	5		3,4	

**Ar_3^{2+} (52 electrons, $D_{\infty h}$)**	σ_g^+	σ_g^-	π_g	δ_g	σ_u^+	σ_u^-	π_u	δ_u
closed orbitals for 10 electrons	1,2,3,4,5,6,7		1		1,2,3,4,5,6		1,2,3	
active in (10,6)	8		2		7		4	
active in (10,7)	8,9		2		7		4	
active in (10,9)	8,9		2,3		7		4	
closed orbitals for 16 electrons	1,2,3,4,5,6,7		1		1,2,3,4,5		1,2	
active in (16,9)	8		2		6,7		3,4	
active in (16,10)	8,9		2		6,7		3,4	

**Xe_3^{2+} (76 electrons, $D_{\infty h}$)**	σ_g^+	σ_g^-	π_g	δ_g	σ_u^+	σ_u^-	π_u	δ_u
closed orbitals for 10 electrons	1,2,3,4,5,6,7		1,2,3	1,2	1,2,3,4,5,6		1,2,3,4	1
active in (10,6)	8		4		7		5	
active in (10,7)	8,9		4		7		5	
active in (10,9)	8,9		4,5		7		5	
closed orbitals for 14 electrons	1,2,3,4,5,6,7		1,2,3	1,2	1,2,3,4,5,6		1,2,3	1
active in (14,9)	8,9		4		7		4,5	
closed orbitals for 16 electrons	1,2,3,4,5,6,7		1,2,3	1,2	1,2,3,4,5		1,2,3	1
active in (16,9)	8		4		6,7		4,5	

active in (16,10)	8,9		4		6,7		4,5	
active in (16,10)’	8		4		6,7,8		4,5	
active in (16,11)	8,9		4		6,7,8		4,5	
active in (16,12)	8,9		4	3	6,7		4,5	
active in (16,12)’	8,9		4,5		6,7		4,5	
active in (16,13)	8,9		4	3	6,7,8		4,5	
active in (16,13)’	8,9		4,5		6,7,8		4,5	

Tab. S2
Definition of the active spaces for Ng_3^{2+}, Ng = Kr, Xe in the $C_{\infty v}$ point group used for 2D scans with variation of both bond lengths.

**Kr_3^{2+} (106 electrons, $C_{\infty v}$)**	σ^+	σ^-	π	δ
closed orbitals	1–21		1,2,3,4,5,6,7,8,9	1,2,3
active in (16,9)	22,23,24		10,11,12	
active in (16,10)	22,23,24,25		10,11,12	

**Xe_3^{2+} (76 electrons, $C_{\infty v}$)**	σ^+	σ^-	π	δ
closed orbitals	1–12		1,2,3,4,5,6	1,2,3
active in (16,9)	13,14,15		7,8,9	
active in (16,10)	13,14,15,16		7,8,9	

Tab. S3

Electronic states included in the CAS calculation for Ng_2^{2+} (Ng = Ar, Kr, Xe) and Ng_3^{2+} (Ng = Ar, Xe) in the $D_{\infty h}$ point group used for 1D scans. The calculated states are the same for all used active spaces. For the electronic state analysis of Ar_2^{2+} in Fig. 3a), MRCI calculations were performed for all electronic states. For all other 1D scans, MRCI calculations were performed only for the lowest-lying electronic state.

**Ar_2^{2+} electronic state analysis ($D_{\infty h}$)**	Σ_g^+	Σ_g^-	Π_g	Δ_g	Σ_u^+	Σ_u^-	Π_u	Δ_u
CAS singlet states	1,2		1	1		1	1	
CAS triplet states		1	1		1,2		1	1

**Ng_2^{2+} 1D Scans (Ng = Ar, Kr, Xe) ($D_{\infty h}$)**	Σ_g^+	Σ_g^-	Π_g	Δ_g	Σ_u^+	Σ_u^-	Π_u	Δ_u
CAS singlet states	1		1				1	
CAS triplet states			1				1	

**Ng_3^{2+} 1D Scans (Ng = Ar, Xe) ($D_{\infty h}$)**	Σ_g^+	Σ_g^-	Π_g	Δ_g	Σ_u^+	Σ_u^-	Π_u	Δ_u
CAS singlet states	1		1				1	
CAS triplet states			1		1		1	

Tab. S4

Electronic states included in the CAS calculation for Ng_3^{2+} (Ng = Kr, Xe) in the $C_{\infty v}$ point group, which was used for 2D scans. The calculated states are the same for all used active spaces. For all 2D scans, MRCI calculations were performed only for the lowest-lying electronic state.

**Ng_3^{2+} (Ng = Kr, Xe) ($C_{\infty v}$)**	Σ^+	Σ^-	Π	Δ
CAS singlet states	1		1,2	
CAS triplet states	1		1,2	

Tab. S5

Dissociation energies of Ng_2^+ in eV.

Ng	This work	Ref. a	Ref. b
Ar	1.31	1.39	
Kr	1.28	1.35	1.32
Xe	1.23		1.25

Ref. a R. Mastalerz, O. Zehnder, M. Reiher, F. Merkt, J. Chem. Theory Comput. 2012, 8 (10), 3671–3685, https://doi.org/10.1021/ct300078m

Ref. b Y.N. Liang, F. Wang, J.W. Guo, J. Chem. Phys. 138, 094319 (2013), https://doi.org/10.1063/1.4792435

Tab. S6 Potential energy curves calculated employing the def2QZVPPD basis set (Å / Hartree).

Ar_2^{2+}, CCSD(T)
1.4 -0.10524530320D+04
1.45 -0.10525452852D+04
1.5 -0.10526171960D+04
1.55 -0.10526729447D+04
1.6 -0.10527158330D+04
1.65 -0.10527484799D+04
1.7 -0.10527729730D+04
1.75 -0.10527909844D+04
1.8 -0.10528038607D+04
1.85 -0.10528126919D+04
1.9 -0.10528183641D+04
2 -0.10528229946D+04
2.015 -0.10528231285D+04
2.02 -0.10528231481D+04
2.025 -0.10528231559D+04
2.026 -0.10528231561D+04
2.027 -0.10528231558D+04
2.028 -0.10528231551D+04
2.03 -0.10528231523D+04
2.035 -0.10528231376D+04
2.05 -0.10528230317D+04
2.075 -0.10528226689D+04
2.1 -0.10528221104D+04
2.15 -0.10528205575D+04
2.2 -0.10528186396D+04
2.25 -0.10528165727D+04
2.3 -0.10528145300D+04
2.35 -0.10528126479D+04
2.4 -0.10528110314D+04
2.45 -0.10528097579D+04
2.5 -0.10528088810D+04
2.55 -0.10528084335D+04
2.6 -0.10528084301D+04
2.65 -0.10528088706D+04
2.7 -0.10528097401D+04
2.75 -0.10528110149D+04
2.8 -0.10528126631D+04
2.85 -0.10528146469D+04
2.9 -0.10528169248D+04
3 -0.10528221913D+04
3.05 -0.10528250954D+04
3.1 -0.10528281270D+04
3.15 -0.10528312500D+04
3.2 -0.10528344319D+04
3.25 -0.10528376441D+04
3.3 -0.10528408617D+04
3.35 -0.10528440638D+04
3.4 -0.10528472329D+04
3.45 -0.10528503550D+04
3.5 -0.10528534189D+04
3.55 -0.10528564162D+04
3.6 -0.10528593408D+04
3.65 -0.10528621883D+04
3.7 -0.10528649561D+04
3.75 -0.10528676430D+04
3.8 -0.10528702488D+04
3.85 -0.10528727742D+04
3.9 -0.10528752205D+04
4 -0.10528798835D+04
4.05 -0.10528821049D+04
4.1 -0.10528842564D+04
4.15 -0.10528863407D+04
4.2 -0.10528883607D+04
4.25 -0.10528903190D+04
4.3 -0.10528922185D+04
4.35 -0.10528940617D+04
4.4 -0.10528958511D+04
4.45 -0.10528975898D+04
4.5 -0.10528992795D+04
4.55 -0.10529009224D+04
4.6 -0.10529025212D+04
4.65 -0.10529040775D+04
4.7 -0.10529055934D+04
4.75 -0.10529070705D+04
4.8 -0.10529085107D+04
4.85 -0.10529099155D+04
4.9 -0.10529112864D+04

Ar_3^{2+}, CCSD(T)
1.4 -0.15788919320D+04
1.5 -0.15792904334D+04
1.6 -0.15795567101D+04
1.7 -0.15797322398D+04
1.8 -0.15798452073D+04
1.9 -0.15799153269D+04
2 -0.15799565869D+04
2.1 -0.15799789426D+04
2.2 -0.15799894449D+04
2.3 -0.15799930281D+04
2.325 -0.15799932663D+04
2.335 -0.15799933103D+04
2.34 -0.15799933225D+04
2.345 -0.15799933286D+04
2.346 -0.15799933291D+04
2.347 -0.15799933294D+04
2.348 -0.15799933295D+04
2.349 -0.15799933293D+04
2.35 -0.15799933289D+04
2.351 -0.15799933283D+04
2.352 -0.15799933275D+04
2.355 -0.15799933236D+04
2.36 -0.15799933131D+04
2.365 -0.15799932975D+04
2.375 -0.15799932523D+04
2.4 -0.15799930690D+04
2.5 -0.15799918036D+04
2.6 -0.15799906397D+04
2.625 -0.15799904707D+04
2.65 -0.15799903695D+04
2.655 -0.15799903581D+04
2.66 -0.15799903496D+04
2.665 -0.15799903443D+04
2.666 -0.15799903436D+04
2.667 -0.15799903430D+04
2.668 -0.15799903426D+04
2.669 -0.15799903423D+04
2.67 -0.15799903421D+04
2.671 -0.15799903420D+04
2.672 -0.15799903421D+04
2.673 -0.15799903423D+04
2.674 -0.15799903426D+04
2.675 -0.15799903431D+04
2.68 -0.15799903472D+04
2.685 -0.15799903547D+04
2.69 -0.15799903653D+04
2.695 -0.15799903794D+04
2.7 -0.15799903968D+04
2.75 -0.15799907624D+04
2.8 -0.15799914906D+04
2.9 -0.15799940727D+04
3 -0.15799981396D+04
3.1 -0.15800035942D+04
3.2 -0.15800102843D+04
3.3 -0.15800180201D+04
3.4 -0.15800265903D+04
3.5 -0.15800357745D+04
3.6 -0.15800453634D+04
3.7 -0.15800551630D+04
3.8 -0.15800650100D+04
3.9 -0.15800747667D+04
4 -0.15800843275D+04
4.1 -0.15800936092D+04
4.2 -0.15801025498D+04
4.3 -0.15801111068D+04
4.4 -0.15801192508D+04
4.5 -0.15801269655D+04
4.6 -0.15801342430D+04
4.7 -0.15801410827D+04

Kr_2^{2+}, CCSD(T)
1.6 -0.55036982187D+04
1.65 -0.55037990761D+04
1.7 -0.55038799101D+04
1.75 -0.55039444278D+04
1.8 -0.55039956501D+04
1.85 -0.55040360394D+04
1.9 -0.55040676063D+04
1.95 -0.55040919947D+04
2 -0.55041105527D+04
2.05 -0.55041243881D+04
2.1 -0.55041344135D+04
2.15 -0.55041413825D+04
2.2 -0.55041459180D+04
2.25 -0.55041485356D+04
2.28 -0.55041493668D+04
2.29 -0.55041495388D+04
2.3 -0.55041496630D+04
2.31 -0.55041497419D+04
2.315 -0.55041497652D+04
2.318 -0.55041497741D+04
2.319 -0.55041497763D+04
2.32 -0.55041497781D+04
2.321 -0.55041497794D+04
2.322 -0.55041497804D+04
2.323 -0.55041497810D+04
2.324 -0.55041497811D+04
2.325 -0.55041497809D+04
2.326 -0.55041497803D+04
2.327 -0.55041497793D+04
2.328 -0.55041497779D+04
2.33 -0.55041497740D+04
2.34 -0.55041497319D+04
2.35 -0.55041496542D+04
2.4 -0.55041488041D+04
2.45 -0.55041473575D+04
2.5 -0.55041455181D+04
2.55 -0.55041434548D+04
2.6 -0.55041413072D+04
2.65 -0.55041391898D+04
2.7 -0.55041371953D+04
2.75 -0.55041353974D+04
2.8 -0.55041338527D+04
2.85 -0.55041326032D+04
2.9 -0.55041316776D+04
3 -0.55041308517D+04
3.05 -0.55041309551D+04
3.1 -0.55041313910D+04

3.15 -0.55041321427D+04
3.2 -0.55041331884D+04
3.25 -0.55041345027D+04
3.3 -0.55041360577D+04
3.35 -0.55041378240D+04
3.4 -0.55041397722D+04
3.45 -0.55041418728D+04
3.5 -0.55041440976D+04
3.55 -0.55041464201D+04
3.6 -0.55041488156D+04
3.65 -0.55041512618D+04
3.7 -0.55041537389D+04
3.75 -0.55041562293D+04
3.8 -0.55041587180D+04
3.85 -0.55041611917D+04
3.9 -0.55041636415D+04
4 -0.55041684322D+04
4.05 -0.55041707616D+04
4.1 -0.55041730411D+04
4.15 -0.55041752681D+04
4.2 -0.55041774406D+04
4.25 -0.55041795577D+04
4.3 -0.55041816190D+04
4.35 -0.55041836246D+04
4.4 -0.55041855752D+04
4.45 -0.55041874718D+04
4.5 -0.55041893156D+04
4.55 -0.55041911072D+04
4.6 -0.55041928496D+04
4.65 -0.55041945439D+04
4.7 -0.55041961909D+04
4.75 -0.55041977931D+04
4.8 -0.55041993525D+04
4.85 -0.55042008700D+04
4.9 -0.55042023478D+04

Kr_3^{2+}, CCSD(T)
2 -0.82567115943D+04
2.1 -0.82567961708D+04
2.2 -0.82568486531D+04
2.3 -0.82568793329D+04
2.4 -0.82568954963D+04
2.5 -0.82569023151D+04
2.525 -0.82569030158D+04
2.55 -0.82569034124D+04
2.56 -0.82569034951D+04
2.565 -0.82569035217D+04
2.57 -0.82569035386D+04
2.571 -0.82569035409D+04
2.572 -0.82569035428D+04
2.573 -0.82569035444D+04
2.574 -0.82569035455D+04
2.575 -0.82569035464D+04
2.576 -0.82569035468D+04
2.577 -0.82569035469D+04
2.578 -0.82569035467D+04
2.579 -0.82569035461D+04
2.58 -0.82569035451D+04
2.585 -0.82569035352D+04
2.59 -0.82569035169D+04
2.6 -0.82569034562D+04
2.625 -0.82569031769D+04
2.65 -0.82569027397D+04
2.7 -0.82569015051D+04
2.8 -0.82568982505D+04
2.9 -0.82568948890D+04

Xe_2^{2+}, CCSD(T)
2 -0.65664054933D+03
2.05 -0.65669685583D+03
2.1 -0.65674279952D+03
2.15 -0.65678006738D+03
2.2 -0.65681008008D+03
2.25 -0.65683403418D+03
2.3 -0.65685293782D+03
2.35 -0.65686764100D+03
2.4 -0.65687886092D+03
2.45 -0.65688720329D+03
2.5 -0.65689318002D+03
2.55 -0.65689722411D+03
2.6 -0.65689970193D+03
2.65 -0.65690092367D+03
2.66 -0.65690104114D+03
2.67 -0.65690112089D+03
2.68 -0.65690116476D+03
2.685 -0.65690117379D+03
2.686 -0.65690117459D+03
2.687 -0.65690117506D+03
2.688 -0.65690117521D+03
2.689 -0.65690117502D+03
2.69 -0.65690117451D+03
2.691 -0.65690117368D+03
2.692 -0.65690117252D+03
2.693 -0.65690117104D+03
2.694 -0.65690116925D+03
2.695 -0.65690116713D+03
2.7 -0.65690115186D+03
2.75 -0.65690060873D+03
2.8 -0.65689948222D+03
2.85 -0.65689793143D+03
2.9 -0.65689608971D+03
3 -0.65689196673D+03
3.05 -0.65688985864D+03
3.1 -0.65688781084D+03
3.15 -0.65688587664D+03
3.2 -0.65688409893D+03
3.25 -0.65688251147D+03
3.3 -0.65688113987D+03
3.35 -0.65688000249D+03
3.4 -0.65687911154D+03
3.45 -0.65687847252D+03
3.5 -0.65687808670D+03
3.55 -0.65687795058D+03
3.6 -0.65687805680D+03
3.65 -0.65687839470D+03
3.7 -0.65687895097D+03
3.75 -0.65687971023D+03
3.8 -0.65688065556D+03
3.85 -0.65688176906D+03
3.9 -0.65688303231D+03
4 -0.65688593443D+03
4.05 -0.65688753751D+03
4.1 -0.65688921948D+03
4.15 -0.65689096447D+03
4.2 -0.65689275771D+03
4.25 -0.65689458710D+03
4.3 -0.65689643964D+03
4.35 -0.65689830509D+03
4.4 -0.65690017414D+03
4.45 -0.65690203869D+03
4.5 -0.65690389181D+03
4.55 -0.65690572765D+03
4.6 -0.65690754134D+03
4.65 -0.65690932890D+03
4.7 -0.65691108716D+03
4.75 -0.65691281364D+03
4.8 -0.65691450649D+03
4.85 -0.65691616446D+03
4.9 -0.65691778660D+03
5 -0.65692092197D+03
5.05 -0.65692243521D+03
5.1 -0.65692391256D+03
5.15 -0.65692535454D+03
5.2 -0.65692676185D+03
5.25 -0.65692813526D+03
5.3 -0.65692947564D+03
5.35 -0.65693078389D+03
5.4 -0.65693206108D+03
5.45 -0.65693330818D+03
5.5 -0.65693452596D+03
5.55 -0.65693571553D+03
5.6 -0.65693687766D+03
5.65 -0.65693801398D+03
5.7 -0.65693912470D+03
5.75 -0.65694021099D+03
5.8 -0.65694127374D+03
5.85 -0.65694231432D+03
5.9 -0.65694333204D+03

Xe_3^{2+}, CCSD(T)
2.6 -0.98591593807D+03
2.7 -0.98593437204D+03
2.8 -0.98594336311D+03
2.86 -0.98594560290D+03
2.88 -0.98594595053D+03
2.9 -0.98594612862D+03
2.91 -0.98594615922D+03
2.911 -0.98594616025D+03
2.912 -0.98594616091D+03
2.913 -0.98594616122D+03
2.914 -0.98594616117D+03
2.915 -0.98594616076D+03
2.916 -0.98594616000D+03
2.917 -0.98594615889D+03
2.918 -0.98594615743D+03
2.919 -0.98594615562D+03
2.92 -0.98594615346D+03
2.925 -0.98594613753D+03
2.93 -0.98594611322D+03
2.94 -0.98594604032D+03
2.96 -0.98594580352D+03
2.98 -0.98594545645D+03
3 -0.98594501164D+03
3.1 -0.98594170620D+03
3.2 -0.98593741854D+03

Ar_2^{2+} MRCI(10,11) $1^1\Sigma_g^+$ (Fig. 3a)
1.70 -1052.7410696827
1.75 -1052.7585897017
1.80 -1052.7709929080
1.85 -1052.7793769570
1.90 -1052.7846354315
1.95 -1052.7874982095
2.00 -1052.7885631445
2.05 -1052.7883207087
2.10 -1052.7871730892
2.15 -1052.7854490522
2.20 -1052.7834154181
2.25 -1052.7812859800
2.30 -1052.7792285515
2.35 -1052.7773706897
2.40 -1052.7758046379
2.45 -1052.7745919416
2.50 -1052.7737680366
2.55 -1052.7733471352
2.60 -1052.7733278894
2.65 -1052.7737014506
2.70 -1052.7744695260

2.75	-1052.7757195112
2.80	-1052.7779436371
2.85	-1052.7812893161
2.90	-1052.7848878939
2.95	-1052.7882180535
3.00	-1052.7913105813
3.05	-1052.7941987821
3.10	-1052.7969108178
3.15	-1052.7994704504
3.20	-1052.8018976904
3.25	-1052.8042093591
3.30	-1052.8064195710
3.35	-1052.8085401548
3.40	-1052.8105810119
3.45	-1052.8125504299
3.50	-1052.8144553471
3.55	-1052.8163032083
3.60	-1052.8181040698
3.65	-1052.8198545866
3.70	-1052.8215583234
3.75	-1052.8232183214
3.80	-1052.8248371930
3.85	-1052.8264171978
3.90	-1052.8279603072
3.95	-1052.8294682549
4.00	-1052.8309425801

Ar_2^{2+} MRCI(10,11) $^1\Delta_g$ (Fig. 3a)

1.70	-1052.3287029431
1.75	-1052.3867370054
1.80	-1052.4375140863
1.85	-1052.4819916859
1.90	-1052.5209922142
1.95	-1052.5552199285
2.00	-1052.5852770938
2.05	-1052.6116798421
2.10	-1052.6348735022
2.15	-1052.6552458644
2.20	-1052.6731379453
2.25	-1052.6888519993
2.30	-1052.7026572876
2.35	-1052.7147941403
2.40	-1052.7254768859
2.45	-1052.7348962190
2.50	-1052.7432211148
2.55	-1052.7506006259
2.60	-1052.7571655877
2.65	-1052.7630302766
2.70	-1052.7682940225
2.75	-1052.7730427596
2.80	-1052.7773505247
2.85	-1052.7812808033
2.90	-1052.7847699364
2.95	-1052.7880728141
3.00	-1052.7911673757
3.05	-1052.7940678236
3.10	-1052.7967955878
3.15	-1052.7993717082
3.20	-1052.8018150288
3.25	-1052.8041418781
3.30	-1052.8063661910
3.35	-1052.8084997652
3.40	-1052.8105525423
3.45	-1052.8125328810
3.50	-1052.8144478024
3.55	-1052.8163015822
3.60	-1052.8180940262
3.65	-1052.8198368058
3.70	-1052.8215334189
3.75	-1052.8231868472
3.80	-1052.8247996499
3.85	-1052.8263740396
3.90	-1052.8279119458
3.95	-1052.8294150649
4.00	-1052.8308849033

Ar_2^{2+} MRCI(10,11) $2^1\Sigma_g^+$ (Fig. 3a)

1.70	-1052.3088667270
1.75	-1052.3688398654
1.80	-1052.4216125845
1.85	-1052.4680699430
1.90	-1052.5089671426
1.95	-1052.5449543487
2.00	-1052.5765975378
2.05	-1052.6043949072
2.10	-1052.6287898908
2.15	-1052.6501801502
2.20	-1052.6689237679
2.25	-1052.6853433843
2.30	-1052.6997291466
2.35	-1052.7123409903
2.40	-1052.7234106126
2.45	-1052.7331433457
2.50	-1052.7417198659
2.55	-1052.7492976582
2.60	-1052.7560117042
2.65	-1052.7619726408
2.70	-1052.7672545981
2.75	-1052.7718254343
2.80	-1052.7752323231
2.85	-1052.7773553702
2.90	-1052.7792000637
2.95	-1052.7810918752
3.00	-1052.7830700006
3.05	-1052.7851261102
3.10	-1052.7872429010
3.15	-1052.7894023946
3.20	-1052.7915881918
3.25	-1052.7937860747
3.30	-1052.7959840530
3.35	-1052.7981722097
3.40	-1052.8003424749
3.45	-1052.8024883872
3.50	-1052.8046048620
3.55	-1052.8066879817
3.60	-1052.8087348082
3.65	-1052.8107432182
3.70	-1052.8127117617
3.75	-1052.8146395388
3.80	-1052.8165260966
3.85	-1052.8183713406
3.90	-1052.8201754628
3.95	-1052.8219388775
4.00	-1052.8236621734

Ar_2^{2+} MRCI(10,11) $^1\Pi_u$ (Fig. 3a)

1.70	-1052.5287546961
1.75	-1052.5664368984
1.80	-1052.5977691163
1.85	-1052.6238069237
1.90	-1052.6454520680
1.95	-1052.6634767776
2.00	-1052.6785408008
2.05	-1052.6912042338
2.10	-1052.7019379726
2.15	-1052.7111329022
2.20	-1052.7191085465
2.25	-1052.7261215859
2.30	-1052.7323744396
2.35	-1052.7380238613
2.40	-1052.7431893060
2.45	-1052.7479607240
2.50	-1052.7524054595
2.55	-1052.7565740334
2.60	-1052.7605047513
2.65	-1052.7642272041
2.70	-1052.7677648193
2.75	-1052.7711366497
2.80	-1052.7743586045
2.85	-1052.7774442528
2.90	-1052.7804054185
2.95	-1052.7832524624
3.00	-1052.7859945822
3.05	-1052.7886399675
3.10	-1052.7911959228
3.15	-1052.7936689608
3.20	-1052.7960648765
3.25	-1052.7983888149
3.30	-1052.8006453287
3.35	-1052.8028384405
3.40	-1052.8049717004
3.45	-1052.8070482461
3.50	-1052.8090708587
3.55	-1052.8110420164
3.60	-1052.8129639424
3.65	-1052.8148386464
3.70	-1052.8166679616
3.75	-1052.8184535727
3.80	-1052.8201970419
3.85	-1052.8218998268
3.90	-1052.8235632979
3.95	-1052.8251887491
4.00	-1052.8267774091

Ar_2^{2+} MRCI(10,11) $^1\Pi_g$ (Fig. 3a)

1.70	-1052.3629538808
1.75	-1052.4208468713
1.80	-1052.4706327545
1.85	-1052.5134229752
1.90	-1052.5501930729
1.95	-1052.5817969313
2.00	-1052.6089791346
2.05	-1052.6323860323
2.10	-1052.6525759551
2.15	-1052.6700289656
2.20	-1052.6851561066
2.25	-1052.6983081536
2.30	-1052.7097837115
2.35	-1052.7198365610
2.40	-1052.7286821743
2.45	-1052.7365033847
2.50	-1052.7434552594
2.55	-1052.7496692446
2.60	-1052.7552566844
2.65	-1052.7603118055
2.70	-1052.7649142536
2.75	-1052.7691312524
2.80	-1052.7730194469
2.85	-1052.7766265444
2.90	-1052.7799925910
2.95	-1052.7831512083
3.00	-1052.7861305806
3.05	-1052.7889543141
3.10	-1052.7916421664
3.15	-1052.7942106622
3.20	-1052.7966736130
3.25	-1052.7990425566
3.30	-1052.8013271250
3.35	-1052.8035353615

3.40	-1052.8056739864
3.45	-1052.8077486278
3.50	-1052.8097640142
3.55	-1052.8117241393
3.60	-1052.8136323994
3.65	-1052.8154917064
3.70	-1052.8173045818
3.75	-1052.8190732309
3.80	-1052.8207996043
3.85	-1052.8224854462
3.90	-1052.8241323333
3.95	-1052.8257417043
4.00	-1052.8273148853

Ar_2^{2+} MRCI(10,11) $^1\Sigma_u^-$ (Fig. 3a)

1.70	-1052.2320757381
1.75	-1052.3075668604
1.80	-1052.3733131124
1.85	-1052.4304230429
1.90	-1052.4799318299
1.95	-1052.5227841521
2.00	-1052.5598322993
2.05	-1052.5918384698
2.10	-1052.6194793468
2.15	-1052.6433515919
2.20	-1052.6639785605
2.25	-1052.6818171584
2.30	-1052.6972647543
2.35	-1052.7106658267
2.40	-1052.7223181713
2.45	-1052.7324785725
2.50	-1052.7413679191
2.55	-1052.7491757768
2.60	-1052.7560644506
2.65	-1052.7621726134
2.70	-1052.7676183455
2.75	-1052.7725021177
2.80	-1052.7769091233
2.85	-1052.7809114566
2.90	-1052.7845699977
2.95	-1052.7879360580
3.00	-1052.7910528056
3.05	-1052.7939565020
3.10	-1052.7966775645
3.15	-1052.7992414732
3.20	-1052.8016695457
3.25	-1052.8039795948
3.30	-1052.8061864850
3.35	-1052.8083026096
3.40	-1052.8103382918
3.45	-1052.8123021316
3.50	-1052.8142012960
3.55	-1052.8160417673
3.60	-1052.8178285517
3.65	-1052.8195658537
3.70	-1052.8212572215
3.75	-1052.8229056666
3.80	-1052.8245137629
3.85	-1052.8260837272
3.90	-1052.8276174859
3.95	-1052.8291167272
4.00	-1052.8305829463

Ar_2^{2+} MRCI(10,11) $^3\Pi_u$ (Fig. 3a)

1.70	-1052.5646891449
1.75	-1052.6009502941
1.80	-1052.6307794452
1.85	-1052.6552368711
1.90	-1052.6752278312
1.95	-1052.6915290306
2.00	-1052.7048075927
2.05	-1052.7156352506
2.10	-1052.7244994637
2.15	-1052.7318124627
2.20	-1052.7379189028
2.25	-1052.7431026250
2.30	-1052.7475929711
2.35	-1052.7515710203
2.40	-1052.7551759641
2.45	-1052.7585116367
2.50	-1052.7616530255
2.55	-1052.7646524529
2.60	-1052.7675451337
2.65	-1052.7703538915
2.70	-1052.7730929420
2.75	-1052.7757707731
2.80	-1052.7783922455
2.85	-1052.7809600231
2.90	-1052.7834756073
2.95	-1052.7859398381
3.00	-1052.7883533306
3.05	-1052.7907166556
3.10	-1052.7930304339
3.15	-1052.7952953674
3.20	-1052.7975122355
3.25	-1052.7996818757
3.30	-1052.8018051581
3.35	-1052.8038829664
3.40	-1052.8059161829
3.45	-1052.8079056825
3.50	-1052.8098523294
3.55	-1052.8117569801
3.60	-1052.8136204866
3.65	-1052.8154436996
3.70	-1052.8172274720
3.75	-1052.8189726596
3.80	-1052.8206801223
3.85	-1052.8223507231
3.90	-1052.8239853274
3.95	-1052.8255847996
4.00	-1052.8271500028

Ar_2^{2+} MRCI(10,11) $^3\Sigma_g^-$ (Fig. 3a)

1.70	-1052.3525196462
1.75	-1052.4085154042
1.80	-1052.4571251599
1.85	-1052.4993689198
1.90	-1052.5361395507
1.95	-1052.5682122843
2.00	-1052.5962520290
2.05	-1052.6208221401
2.10	-1052.6423959019
2.15	-1052.6613701274
2.20	-1052.6780792437
2.25	-1052.6928081899
2.30	-1052.7058030122
2.35	-1052.7172788567
2.40	-1052.7274256104
2.45	-1052.7364117460
2.50	-1052.7443869614
2.55	-1052.7514840895
2.60	-1052.7578206112
2.65	-1052.7634999710
2.70	-1052.7686127859
2.75	-1052.7732380991
2.80	-1052.7774444121
2.85	-1052.7812908739
2.90	-1052.7848283200
2.95	-1052.7881002713
3.00	-1052.7911438551
3.05	-1052.7939906482
3.10	-1052.7966674350
3.15	-1052.7991968773
3.20	-1052.8015981057
3.25	-1052.8038872358
3.30	-1052.8060778146
3.35	-1052.8081812120
3.40	-1052.8102069564
3.45	-1052.8121630301
3.50	-1052.8140561201
3.55	-1052.8158918353
3.60	-1052.8176748918
3.65	-1052.8194092681
3.70	-1052.8210983367
3.75	-1052.8227449722
3.80	-1052.8243516421
3.85	-1052.8259204801
3.90	-1052.8274533478
3.95	-1052.8289518830
4.00	-1052.8304175415

Ar_2^{2+} MRCI(10,11) $1^3\Sigma_u^+$ (Fig. 3a)

1.70	-1052.3528837113
1.75	-1052.4055456893
1.80	-1052.4515693090
1.85	-1052.4917965296
1.90	-1052.5269719773
1.95	-1052.5577514084
2.00	-1052.5847155173
2.05	-1052.6083860851
2.10	-1052.6292517242
2.15	-1052.6478328929
2.20	-1052.6648455126
2.25	-1052.6809977620
2.30	-1052.6962278619
2.35	-1052.7097752688
2.40	-1052.7215582336
2.45	-1052.7318351060
2.50	-1052.7408282315
2.55	-1052.7487285174
2.60	-1052.7556994956
2.65	-1052.7618809154
2.70	-1052.7673919240
2.75	-1052.7723338604
2.80	-1052.7767927213
2.85	-1052.7808413172
2.90	-1052.7845411645
2.95	-1052.7879441392
3.00	-1052.7910939095
3.05	-1052.7940271790
3.10	-1052.7967747559
3.15	-1052.7993624649
3.20	-1052.8018119275
3.25	-1052.8041412245
3.30	-1052.8063654565
3.35	-1052.8084972249
3.40	-1052.8105470369
3.45	-1052.8125236546
3.50	-1052.8144343887
3.55	-1052.8162853490
3.60	-1052.8180816548
3.65	-1052.8198276111
3.70	-1052.8215268557
3.75	-1052.8231824797
3.80	-1052.8247971282
3.85	-1052.8263730811
3.90	-1052.8279123213

3.95	-1052.8294165872
4.00	-1052.8308874186

Ar_2^{2+} MRCI(10,11) $^3\Delta_u$ (Fig. 3a)

1.70	-1052.2274979128
1.75	-1052.3034030804
1.80	-1052.3695671900
1.85	-1052.4270719514
1.90	-1052.4769466328
1.95	-1052.5201347246
2.00	-1052.5574890618
2.05	-1052.5897732056
2.10	-1052.6176654411
2.15	-1052.6417643371
2.20	-1052.6625951087
2.25	-1052.6806165524
2.30	-1052.6959570608
2.35	-1052.7092804584
2.40	-1052.7209872787
2.45	-1052.7312475036
2.50	-1052.7402505692
2.55	-1052.7481731339
2.60	-1052.7551718921
2.65	-1052.7613830538
2.70	-1052.7669239061
2.75	-1052.7718947843
2.80	-1052.7763811618
2.85	-1052.7804556009
2.90	-1052.7841795291
2.95	-1052.7876048269
3.00	-1052.7907752200
3.05	-1052.7937274989
3.10	-1052.7964925731
3.15	-1052.7990963742
3.20	-1052.8015606305
3.25	-1052.8039035260
3.30	-1052.8061402596
3.35	-1052.8082835247
3.40	-1052.8103439139
3.45	-1052.8123302684
3.50	-1052.8142499712
3.55	-1052.8161091984
3.60	-1052.8179131298
3.65	-1052.8196661256
3.70	-1052.8213718738
3.75	-1052.8230335112
3.80	-1052.8246537242
3.85	-1052.8262348308
3.90	-1052.8277788479
3.95	-1052.8292875452
4.00	-1052.8307624909

Ar_2^{2+} MRCI(10,11) $2^3\Sigma_u^+$ (Fig. 3a)

1.70	-1052.2231524896
1.75	-1052.2993278587
1.80	-1052.3658078181
1.85	-1052.4236062198
1.90	-1052.4737386163
1.95	-1052.5171414538
2.00	-1052.5546582616
2.05	-1052.5870333847
2.10	-1052.6148979327
2.15	-1052.6387172057
2.20	-1052.6586394106
2.25	-1052.6747112542
2.30	-1052.6879226513
2.35	-1052.6992874094
2.40	-1052.7092807484
2.45	-1052.7181601242
2.50	-1052.7261046904
2.55	-1052.7332549060
2.60	-1052.7397262949
2.65	-1052.7456156903
2.70	-1052.7510049518
2.75	-1052.7559634522
2.80	-1052.7605500332
2.85	-1052.7648145875
2.90	-1052.7687993942
2.95	-1052.7725402605
3.00	-1052.7760674979
3.05	-1052.7794067634
3.10	-1052.7825797766
3.15	-1052.7856049283
3.20	-1052.7884977982
3.25	-1052.7912715938
3.30	-1052.7939375199
3.35	-1052.7965050980
3.40	-1052.7989824362
3.45	-1052.8013764636
3.50	-1052.8036931279
3.55	-1052.8059375652
3.60	-1052.8081142419
3.65	-1052.8102270726
3.70	-1052.8122795180
3.75	-1052.8142746631
3.80	-1052.8162152824
3.85	-1052.8181038907
3.90	-1052.8199427855
3.95	-1052.8217340789
4.00	-1052.8234797263

Ar_2^{2+} MRCI(10,11) $^3\Pi_g$ (Fig. 3a)

1.70	-1052.3953224073
1.75	-1052.4498180420
1.80	-1052.4964147855
1.85	-1052.5362053944
1.90	-1052.5701699710
1.95	-1052.5991712162
2.00	-1052.6239608328
2.05	-1052.6451888511
2.10	-1052.6634135339
2.15	-1052.6791112238
2.20	-1052.6926858866
2.25	-1052.7044782390
2.30	-1052.7147743902
2.35	-1052.7238138090
2.40	-1052.7317965368
2.45	-1052.7388895430
2.50	-1052.7452322000
2.55	-1052.7509409117
2.60	-1052.7561129732
2.65	-1052.7608297644
2.70	-1052.7651593869
2.75	-1052.7691588194
2.80	-1052.7728758075
2.85	-1052.7763502465
2.90	-1052.7796155294
2.95	-1052.7826995876
3.00	-1052.7856257820
3.05	-1052.7884136542
3.10	-1052.7910795546
3.15	-1052.7936371661
3.20	-1052.7960979422
3.25	-1052.7984714733
3.30	-1052.8007657917
3.35	-1052.8029876338
3.40	-1052.8051426590
3.45	-1052.8072356399
3.50	-1052.8092706204
3.55	-1052.8112510519
3.60	-1052.8131799049
3.65	-1052.8150597620
3.70	-1052.8168928938
3.75	-1052.8186813182
3.80	-1052.8204268501
3.85	-1052.8221311387
3.90	-1052.8237956988
3.95	-1052.8254219324
4.00	-1052.8270111494

Ar_2^{2+} MRCI(10,9) (Fig. 3b)

1.80 -1052.761993089881
1.85 -1052.770526846735
1.90 -1052.775925371080
1.95 -1052.778918373158
2.00 -1052.780103564691
2.05 -1052.779971201891
2.10 -1052.778923199274
2.15 -1052.777287898385
2.20 -1052.775331621986
2.25 -1052.773267661953
2.30 -1052.771263675052
2.35 -1052.769447317604
2.40 -1052.767911452522
2.45 -1052.766718514045
2.50 -1052.765904397648
2.55 -1052.765481579943
2.60 -1052.765439688515
2.65 -1052.765732297113
2.70 -1052.766157104581
2.75 -1052.766247862545
2.80 -1052.769254177380
2.85 -1052.772979146996
2.90 -1052.776865464117

Ar_2^{2+} MRCI(10,11) (Fig. 3b)

1.80 -1052.771378488298
1.85 -1052.779748035658
1.90 -1052.784997655463
1.95 -1052.787856634448
1.96 -1052.788196121783
1.97 -1052.788468422517
1.98 -1052.788677592918
1.99 -1052.788827529528
2.00 -1052.788921977954
2.01 -1052.788964536177
2.02 -1052.788958663161
2.03 -1052.788907681651
2.04 -1052.788814783921
2.05 -1052.788683037002
2.06 -1052.788515386214
2.07 -1052.788314659852
2.10 -1052.787540639538
2.15 -1052.785821955141
2.20 -1052.783792108713
2.25 -1052.781663343302
2.30 -1052.779602361999
2.35 -1052.777736270045
2.40 -1052.776157569669
2.45 -1052.774928547663
2.50 -1052.774085426781
2.53 -1052.773771551003
2.54 -1052.773699074436
2.55 -1052.773642607502
2.56 -1052.773602078618
2.57 -1052.773577396931
2.58 -1052.773568454287
2.59 -1052.773575126922
2.60 -1052.773597278331

2.61 -1052.773634760312
2.62 -1052.773687417903
2.65 -1052.773934830172
2.70 -1052.774636757246
2.75 -1052.775700553678
2.80 -1052.777257929781
2.85 -1052.780258542663

Kr_2^{2+} MRCI(10,9) (Fig. 3c)
2.00 -5503.289314917108
2.05 -5503.303580960653
2.10 -5503.313943714934
2.15 -5503.321173273677
2.20 -5503.325907788503
2.25 -5503.328677035060
2.30 -5503.329921440100
2.35 -5503.330007501138
2.40 -5503.329240270715
2.45 -5503.327873425888
2.50 -5503.326117385825
2.55 -5503.324145809499
2.60 -5503.322100775777
2.65 -5503.320096919635
2.70 -5503.318224763739
2.75 -5503.316553458836
2.80 -5503.315133091902
2.85 -5503.313996698811
2.90 -5503.313162008509
2.95 -5503.312632503922
3.00 -5503.312395501462
3.05 -5503.312403741674
3.10 -5503.312451728810
3.15 -5503.313150152443
3.20 -5503.315652729419
3.25 -5503.318359401098
3.30 -5503.321243925398
3.35 -5503.323707805260
3.40 -5503.326016987995
3.45 -5503.328191881616
3.50 -5503.330249920441

Kr_2^{2+} MRCI(10,11) (Fig. 3c)
2.00 -5503.296516434484
2.05 -5503.310650028013
2.10 -5503.320890055592
2.15 -5503.328006439158
2.20 -5503.332637002248
2.25 -5503.335311071543
2.28 -5503.336162637930
2.29 -5503.336339871526
2.30 -5503.336468567377
2.31 -5503.336551433839
2.32 -5503.336591082942
2.33 -5503.336590031303
2.34 -5503.336550706646
2.35 -5503.336475446143
2.36 -5503.336366504998
2.37 -5503.336226055891
2.40 -5503.335636210984
2.45 -5503.334204024425
2.50 -5503.332388833833
2.55 -5503.330363910180
2.60 -5503.328271049764
2.65 -5503.326224713637
2.70 -5503.324315330530
2.75 -5503.322611995867
2.80 -5503.321164778618
2.85 -5503.320006917490
2.90 -5503.319157036314
2.91 -5503.319024774767
2.92 -5503.318905126142
2.93 -5503.318798079154
2.94 -5503.318703611422
2.95 -5503.318621689168
2.96 -5503.318552268928
2.97 -5503.318495299933
2.98 -5503.318450755374
2.99 -5503.318418487467
3.00 -5503.318398522665
3.01 -5503.318390772482
3.02 -5503.318395185368
3.03 -5503.318411716239
3.04 -5503.318440339438
3.05 -5503.318481052585
3.06 -5503.318533886689
3.07 -5503.318598918126
3.08 -5503.318676291385
3.09 -5503.318766237553
3.10 -5503.318869118893
3.15 -5503.319608320324
3.20 -5503.321046224502
3.25 -5503.323941241249
3.30 -5503.326618255685
3.35 -5503.329072763891
3.40 -5503.331373797279
3.45 -5503.333541566675
3.50 -5503.335593378292

Xe_2^{2+} MRCI(10,9) (Fig. 3d)
2.30 -655.871827087046
2.35 -655.887234450119
2.40 -655.899066868394
2.45 -655.907939899221
2.50 -655.914374269250
2.55 -655.918810994485
2.60 -655.921624023770
2.65 -655.923130766456
2.70 -655.923600887165
2.75 -655.923263575871
2.80 -655.922313620544
2.85 -655.920916418630
2.90 -655.919212124640
2.95 -655.917319069036
3.00 -655.915336561781
3.05 -655.913347205818
3.10 -655.911418800646
3.15 -655.909605922857
3.20 -655.907951272030
3.25 -655.906486816430
3.30 -655.905234805174
3.35 -655.904208667346
3.40 -655.903413712161
3.45 -655.902847204868
3.50 -655.902496001699
3.55 -655.902322885828
3.60 -655.902214522639
3.65 -655.902889187688
3.70 -655.904659148047
3.75 -655.906549672352
3.80 -655.908640896213

Xe_2^{2+} MRCI(10,11) (Fig. 3d)
2.30 -655.878486208366
2.35 -655.893775407512
2.40 -655.905495099414
2.45 -655.914261246666
2.50 -655.920594848475
2.55 -655.924937052223
2.60 -655.927661809852
2.65 -655.929086429678
2.66 -655.929239805798
2.67 -655.929354033161
2.68 -655.929430998455
2.69 -655.929472526381
2.70 -655.929480386095
2.71 -655.929456286476
2.72 -655.929401883509
2.73 -655.929318780742
2.74 -655.929208529354
2.75 -655.929072630705
2.80 -655.928057678762
2.85 -655.926600646069
2.90 -655.924841413702
2.95 -655.922898067372
3.00 -655.920869716591
3.05 -655.918838818873
3.10 -655.916873084120
3.15 -655.915027046535
3.20 -655.913343393192
3.25 -655.911854134556
3.30 -655.910581681581
3.35 -655.909539951236
3.40 -655.908735572167
3.45 -655.908169330214
3.50 -655.907838181584
3.51 -655.907799798354
3.52 -655.907770643347
3.53 -655.907750711727
3.54 -655.907740017667
3.55 -655.907738596850
3.56 -655.907746519219
3.57 -655.907763891477
3.58 -655.907790880883
3.59 -655.907827727075
3.60 -655.907874771334
3.65 -655.908288847244
3.70 -655.909238774725
3.75 -655.911185877567
3.80 -655.913410955386

Ar_3^{2+} MRCI(10,6) (Fig. 4a)
2.10 -1579.844474736605
2.15 -1579.851370784125
2.20 -1579.856200756155
2.25 -1579.859533197719
2.30 -1579.861829674435
2.35 -1579.863451875300
2.40 -1579.864668974292
2.45 -1579.865668289507
2.50 -1579.866569980550
2.55 -1579.867443420633
2.60 -1579.868323212138
2.65 -1579.869222425224
2.70 -1579.870142180118
2.75 -1579.871078141621
2.80 -1579.872024292412
2.85 -1579.872974920158
2.90 -1579.873925460959
2.95 -1579.874872704891
3.00 -1579.875814708588
3.05 -1579.876750616267
3.10 -1579.877680387835
3.15 -1579.878604395532
3.20 -1579.879522980431
3.25 -1579.880436195462
3.30 -1579.881343789815
3.35 -1579.882245252655
3.40 -1579.883139898220
3.45 -1579.884026934204
3.50 -1579.884905523952

3.55 -1579.885774832831
3.60 -1579.886634077513
3.65 -1579.887482544623
3.70 -1579.888319622859
3.75 -1579.889144791629
3.80 -1579.889957670341
3.85 -1579.890757856077
3.90 -1579.891545152445
3.95 -1579.892319399953
4.00 -1579.893080510819

Ar_3^{2+} MRCI(10,7) (Fig. 4a)
2.10 -1579.848392680130
2.15 -1579.855049107953
2.20 -1579.859561195614
2.25 -1579.862465976554
2.30 -1579.864237839575
2.35 -1579.865370877595
2.40 -1579.866224789592
2.45 -1579.866975299095
2.50 -1579.867708352443
2.55 -1579.868466675457
2.60 -1579.869267235362
2.65 -1579.870111606487
2.70 -1579.870993357065
2.75 -1579.871903026705
2.80 -1579.872831085709
2.85 -1579.873769346330
2.90 -1579.874711483675
2.95 -1579.875652996546
3.00 -1579.876591061071
3.05 -1579.877524373724
3.10 -1579.878452764813
3.15 -1579.879376438212
3.20 -1579.880295375663
3.25 -1579.881209285362
3.30 -1579.882117669075
3.35 -1579.883019904841
3.40 -1579.883915262471
3.45 -1579.884802932355
3.50 -1579.885682073937
3.55 -1579.886551853667
3.60 -1579.887411491364
3.65 -1579.888260282111
3.70 -1579.889097611755
3.75 -1579.889922970792
3.80 -1579.890735946130
3.85 -1579.891536223666
3.90 -1579.892323577024
3.95 -1579.893097856977
4.00 -1579.893858984399

Ar_3^{2+} MRCI(10,9) (Fig. 4a)
2.10 -1579.855982609667
2.15 -1579.861997027572
2.20 -1579.866009583100
2.25 -1579.868654323991
2.30 -1579.870390973361
2.35 -1579.871562205026
2.40 -1579.872418897005
2.45 -1579.873133705692
2.50 -1579.873814461185
2.55 -1579.874519611561
2.60 -1579.875273715980
2.65 -1579.876080714384
2.70 -1579.876933890094
2.75 -1579.877822651538
2.80 -1579.878736490644
2.85 -1579.879666377979
2.90 -1579.880604866278
2.95 -1579.881546280522
3.00 -1579.882486773652
3.05 -1579.883423957900
3.10 -1579.884356353361
3.15 -1579.885282757625
3.20 -1579.886201226863
3.25 -1579.887105795451
3.30 -1579.887962248894
3.35 -1579.886681925219
3.40 -1579.888114544407
3.45 -1579.889449290525
3.50 -1579.890699431217
3.55 -1579.891876198043
3.60 -1579.892989046228
3.65 -1579.894045925005
3.70 -1579.895053524120
3.75 -1579.896017488872
3.80 -1579.896942588282
3.85 -1579.897832865623
3.90 -1579.898691756375
3.95 -1579.899522186147
4.00 -1579.900326656734

Ar_3^{2+} MRCI(16,9) (Fig. 4a)
2.10 -1579.847089039588
2.15 -1579.853903333019
2.20 -1579.858590558239
2.25 -1579.861727174999
2.30 -1579.863785827398
2.35 -1579.865142902062
2.40 -1579.866084837977
2.45 -1579.866815923070
2.50 -1579.867470102510
2.55 -1579.868125776218
2.60 -1579.868821521379
2.65 -1579.869570265769
2.70 -1579.870370397018
2.75 -1579.871213487585
2.80 -1579.872089063342
2.85 -1579.872987194881
2.90 -1579.873899649740
2.95 -1579.874820227844
3.00 -1579.875744456618
3.05 -1579.876669448807
3.10 -1579.877593332775
3.15 -1579.878514905456
3.20 -1579.879433313109
3.25 -1579.880347817643
3.30 -1579.881257671633
3.35 -1579.882161949359
3.40 -1579.883060077125
3.45 -1579.883950203949
3.50 -1579.884832522862
3.55 -1579.885705676582
3.60 -1579.886568827831
3.65 -1579.887421206745
3.70 -1579.888262157577
3.75 -1579.889134192819
3.80 -1579.889948513033
3.85 -1579.890750570625
3.90 -1579.891539581352
3.95 -1579.892315534283
4.00 -1579.893078624834

Ar_3^{2+} MRCI(16,10) (Fig. 4a)
2.10 -1579.855437265137
2.15 -1579.862191446392
2.20 -1579.866739001741
2.25 -1579.869636301338
2.30 -1579.871347098913
2.35 -1579.872263953547
2.40 -1579.872716423458
2.45 -1579.872963506538
2.50 -1579.873183911379
2.55 -1579.873479982612
2.60 -1579.873895121526
2.65 -1579.874434889970
2.70 -1579.875085097958
2.75 -1579.875824054489
2.80 -1579.876629551804
2.85 -1579.877482184077
2.90 -1579.878366464823
2.95 -1579.879270790772
3.00 -1579.880186882999
3.05 -1579.881109062346
3.10 -1579.882033526433
3.15 -1579.882957739861
3.20 -1579.883879935708
3.25 -1579.884798764094
3.30 -1579.885713071608
3.35 -1579.886621775189
3.40 -1579.887523811727
3.45 -1579.888418137832
3.50 -1579.889303753991
3.55 -1579.890179720257
3.60 -1579.891045192604
3.65 -1579.891899431506
3.70 -1579.892741809432
3.75 -1579.893571818922
3.80 -1579.894389057435
3.85 -1579.895193227449
3.90 -1579.895984122374
3.95 -1579.896761614190
4.00 -1579.897525646989

Kr_3^{2+} MRCI(16,10) (Fig. 4b, full region)

	2.4	2.5	2.6	2.7	2.8	2.9
2.4	-8255.61367985	-8255.61774213	-8255.61869169	-8255.61760595	-8255.61532462	-8255.61252761
2.5	-8255.61774213	-8255.62181199	-8255.62290141	-8255.62205849	-8255.62014107	-8255.61785498
2.6	-8255.61869169	-8255.62290141	-8255.62426683	-8255.62378087	-8255.62227295	-8255.62045836
2.7	-8255.61760595	-8255.62205849	-8255.62378087	-8255.62373226	-8255.62267641	-8255.62128851
2.8	-8255.61532462	-8255.62014107	-8255.62227295	-8255.62267641	-8255.62210951	-8255.62114151
2.9	-8255.61252761	-8255.61785498	-8255.62045836	-8255.62128851	-8255.62114151	-8255.62059328
3	-8255.60975762	-8255.61572544	-8255.61885051	-8255.62009532	-8255.62027701	-8255.62001569
3.1	-8255.60740360	-8255.61407396	-8255.61772036	-8255.61934731	-8255.61980110	-8255.61972308
3.2	-8255.60568985	-8255.61304516	-8255.61716119	-8255.61910679	-8255.61976827	-8255.61981204
3.3	-8255.60469448	-8255.61265801	-8255.61716176	-8255.61934914	-8255.62014978	-8255.62025789
3.4	-8255.60438987	-8255.61285500	-8255.61765647	-8255.62001062	-8255.62088824	-8255.62101041
3.5	-8255.60468708	-8255.61354189	-8255.61855689	-8255.62101298	-8255.62191654	-8255.62201281
3.6	-8255.60547116	-8255.61461384	-8255.61977127	-8255.62227752	-8255.62316809	-8255.62320887
3.7	-8255.60662574	-8255.61597159	-8255.62121539	-8255.62373298	-8255.62458260	-8255.62454734
3.8	-8255.60804717	-8255.61752955	-8255.62281781	-8255.62531904	-8255.62610881	-8255.62598427
3.9	-8255.60964953	-8255.61921873	-8255.62452120	-8255.62698731	-8255.62770533	-8255.62748388
4	-8255.61136593	-8255.62098568	-8255.62628151	-8255.62870048	-8255.62934009	-8255.62901819
4.1	-8255.61314593	-8255.62279073	-8255.62806601	-8255.63043079	-8255.63098906	-8255.63056609

	3	3.1	3.2	3.3	3.4
2.4	-8255.60975762	-8255.60740360	-8255.60568985	-8255.60469448	-8255.60438987
2.5	-8255.61572544	-8255.61407396	-8255.61304516	-8255.61265801	-8255.61285500
2.6	-8255.61885051	-8255.61772036	-8255.61716119	-8255.61716176	-8255.61765647
2.7	-8255.62009532	-8255.61934731	-8255.61910679	-8255.61934914	-8255.62001062
2.8	-8255.62027701	-8255.61980110	-8255.61976827	-8255.62014978	-8255.62088824
2.9	-8255.62001569	-8255.61972308	-8255.61981204	-8255.62025789	-8255.62101041
3	-8255.61969835	-8255.61953752	-8255.61968176	-8255.62013683	-8255.62086020
3.1	-8255.61953752	-8255.61947186	-8255.61963055	-8255.62005539	-8255.62072071
3.2	-8255.61968176	-8255.61963055	-8255.61977245	-8255.62014243	-8255.62073132
3.3	-8255.62013683	-8255.62005539	-8255.62014243	-8255.62043950	-8255.62094134
3.4	-8255.62086020	-8255.62072071	-8255.62073132	-8255.62094134	-8255.62135005
3.5	-8255.62180327	-8255.62158457	-8255.62150517	-8255.62162030	-8255.62193307
3.6	-8255.62291805	-8255.62260488	-8255.62242615	-8255.62244193	-8255.62265807
3.7	-8255.62416037	-8255.62374269	-8255.62345874	-8255.62337285	-8255.62349286
3.8	-8255.62549199	-8255.62496391	-8255.62457169	-8255.62438346	-8255.62440862
3.9	-8255.62688145	-8255.62624026	-8255.62573883	-8255.62544888	-8255.62538108
4	-8255.62830395	-8255.62754927	-8255.62693930	-8255.62654918	-8255.62639073
4.1	-8255.62974062	-8255.62887372	-8255.62815700	-8255.62766894	-8255.62742244

Kr_3^{2+} MRCI(16,10) (Fig. 4b, zoomed region)

	2.826	2.828	2.83	2.832	2.834
3.136	-8255.61976479	-8255.61976549	-8255.61976606	-8255.61976647	-8255.61976672
3.138	-8255.61976384	-8255.61976460	-8255.61976523	-8255.61976570	-8255.61976601
3.14	-8255.61976306	-8255.61976388	-8255.61976457	-8255.61976509	-8255.61976546
3.142	-8255.61976243	-8255.61976333	-8255.61976408	-8255.61976466	-8255.61976508
3.144	-8255.61976201	-8255.61976295	-8255.61976375	-8255.61976439	-8255.61976487
3.146	-8255.61976183	-8255.61976275	-8255.61976360	-8255.61976429	-8255.61976483
3.148	-8255.61976163	-8255.61976270	-8255.61976361	-8255.61976436	-8255.61976495
3.15	-8255.61976170	-8255.61976283	-8255.61976379	-8255.61976459	-8255.61976524
3.152	-8255.61976193	-8255.61976312	-8255.61976414	-8255.61976500	-8255.61976569
3.154	-8255.61976234	-8255.61976358	-8255.61976465	-8255.61976556	-8255.61976632

	2.836	2.838	2.84	2.842	2.844
3.136	-8255.61976683	-8255.61976679	-8255.61976661	-8255.61976629	-8255.61976583
3.138	-8255.61976617	-8255.61976619	-8255.61976606	-8255.61976580	-8255.61976539
3.14	-8255.61976568	-8255.61976575	-8255.61976568	-8255.61976547	-8255.61976512
3.142	-8255.61976536	-8255.61976549	-8255.61976547	-8255.61976531	-8255.61976501
3.144	-8255.61976520	-8255.61976538	-8255.61976542	-8255.61976531	-8255.61976506
3.146	-8255.61976521	-8255.61976545	-8255.61976553	-8255.61976548	-8255.61976529
3.148	-8255.61976539	-8255.61976567	-8255.61976582	-8255.61976581	-8255.61976567
3.15	-8255.61976573	-8255.61976607	-8255.61976626	-8255.61976631	-8255.61976622
3.152	-8255.61976624	-8255.61976663	-8255.61976687	-8255.61976697	-8255.61976693
3.154	-8255.61976691	-8255.61976736	-8255.61976765	-8255.61976780	-8255.61976781

	2.846	2.848	2.85	2.852
3.136	-8255.61976524	-8255.61976453	-8255.61976368	-8255.61976272
3.138	-8255.61976486	-8255.61976419	-8255.61976340	-8255.61976249
3.14	-8255.61976463	-8255.61976402	-8255.61976328	-8255.61976242
3.142	-8255.61976458	-8255.61976402	-8255.61976333	-8255.61976252
3.144	-8255.61976469	-8255.61976418	-8255.61976354	-8255.61976278
3.146	-8255.61976496	-8255.61976450	-8255.61976391	-8255.61976320
3.148	-8255.61976539	-8255.61976498	-8255.61976445	-8255.61976378
3.15	-8255.61976599	-8255.61976563	-8255.61976514	-8255.61976453
3.152	-8255.61976676	-8255.61976644	-8255.61976600	-8255.61976544
3.154	-8255.61976768	-8255.61976742	-8255.61976703	-8255.61976651

Xe_3^{2+} MRCI(16,10) (Fig. 4c, full region)

	2.8	2.9	3	3.1	3.2
2.8	-984.42792134	-984.43012101	-984.43018865	-984.42883391	-984.42660009
2.9	-984.43012101	-984.43228156	-984.43239496	-984.43115825	-984.42911649
3	-984.43018865	-984.43239496	-984.43263568	-984.43158809	-984.42978765
3.1	-984.42883391	-984.43115825	-984.43158809	-984.43078062	-984.42925463
3.2	-984.42660009	-984.42911649	-984.42978765	-984.42925463	-984.42802442
3.3	-984.42391725	-984.42670945	-984.42767250	-984.42743509	-984.42650345
3.4	-984.42113573	-984.42429046	-984.42559185	-984.42566145	-984.42501218
3.5	-984.41853592	-984.42212412	-984.42379340	-984.42416979	-984.42377658
3.6	-984.41632197	-984.42038068	-984.42242068	-984.42308568	-984.42291536
3.7	-984.41461573	-984.41914177	-984.42152745	-984.42244594	-984.42245812
3.8	-984.41346174	-984.41841808	-984.42110438	-984.42223047	-984.42238083
3.9	-984.41284445	-984.41817223	-984.42110443	-984.42238937	-984.42263495
4	-984.41270853	-984.41834035	-984.42146292	-984.42286100	-984.42316380
4.1	-984.41297901	-984.41884867	-984.42211108	-984.42358295	-984.42391168
4.2	-984.41357614	-984.41962461	-984.42298413	-984.42449796	-984.42482824
4.3	-984.41442480	-984.42060287	-984.42402527	-984.42555652	-984.42587030
4.4	-984.41545980	-984.42172807	-984.42518701	-984.42671754	-984.42700206
4.5	-984.41662680	-984.42295515	-984.42643104	-984.42794791	-984.42819462
4.6	-984.41788263	-984.42424850	-984.42772718	-984.42922154	-984.42942509
4.7	-984.41919379	-984.42558068	-984.42905222	-984.43051831	-984.43067571
4.8	-984.42053492	-984.42693102	-984.43038865	-984.43182296	-984.43193293

	3.3	3.4	3.5	3.6
2.8	-984.42391725	-984.42113573	-984.41853592	-984.41632197
2.9	-984.42670945	-984.42429046	-984.42212412	-984.42038068
3	-984.42767250	-984.42559185	-984.42379340	-984.42242068
3.1	-984.42743509	-984.42566145	-984.42416979	-984.42308568
3.2	-984.42650345	-984.42501218	-984.42377658	-984.42291536
3.3	-984.42527610	-984.42404944	-984.42303160	-984.42234093
3.4	-984.42404944	-984.42306157	-984.42223257	-984.42167282
3.5	-984.42303160	-984.42223257	-984.42155763	-984.42110005
3.6	-984.42234093	-984.42167282	-984.42110005	-984.42071335
3.7	-984.42200996	-984.42142453	-984.42090352	-984.42054734
3.8	-984.42201742	-984.42147572	-984.42096876	-984.42060772
3.9	-984.42231878	-984.42178857	-984.42126780	-984.42087700
4	-984.42286324	-984.42231843	-984.42176275	-984.42132488
4.1	-984.42360151	-984.42302200	-984.42241564	-984.42191856
4.2	-984.42448949	-984.42386047	-984.42319215	-984.42262752
4.3	-984.42548939	-984.42480060	-984.42406271	-984.42342511
4.4	-984.42656984	-984.42581465	-984.42500250	-984.42428877
4.5	-984.42770539	-984.42688002	-984.42599109	-984.42519979
4.6	-984.42887576	-984.42797847	-984.42701196	-984.42614291
4.7	-984.43006506	-984.42909567	-984.42805195	-984.42710586
4.8	-984.43126109	-984.43022048	-984.42910077	-984.42807901

Xe_3^{2+} MRCI(16,10) (Fig. 4c, zoomed region)

	3.152	3.154	3.156	3.158	3.16
3.772	-984.42239858	-984.42240011	-984.42240136	-984.42240234	-984.42240305
3.774	-984.42239761	-984.42239920	-984.42240050	-984.42240153	-984.42240229
3.776	-984.42239677	-984.42239844	-984.42239979	-984.42240087	-984.42240168
3.778	-984.42239611	-984.42239782	-984.42239922	-984.42240034	-984.42240120
3.78	-984.42239561	-984.42239735	-984.42239880	-984.42239997	-984.42240087
3.782	-984.42239519	-984.42239702	-984.42239852	-984.42239974	-984.42240069
3.784	-984.42239500	-984.42239684	-984.42239838	-984.42239965	-984.42240065
3.786	-984.42239488	-984.42239680	-984.42239839	-984.42239971	-984.42240075
3.788	-984.42239494	-984.42239690	-984.42239854	-984.42239990	-984.42240100
3.79	-984.42239513	-984.42239715	-984.42239883	-984.42240024	-984.42240138

	3.162	3.164	3.166	3.168	3.17
3.772	-984.42240351	-984.42240370	-984.42240363	-984.42240332	-984.42240275
3.774	-984.42240279	-984.42240303	-984.42240301	-984.42240274	-984.42240222
3.776	-984.42240222	-984.42240251	-984.42240254	-984.42240232	-984.42240184
3.778	-984.42240180	-984.42240213	-984.42240221	-984.42240203	-984.42240160
3.78	-984.42240152	-984.42240190	-984.42240202	-984.42240189	-984.42240151
3.782	-984.42240138	-984.42240181	-984.42240197	-984.42240189	-984.42240155
3.784	-984.42240139	-984.42240186	-984.42240207	-984.42240203	-984.42240174
3.786	-984.42240153	-984.42240205	-984.42240231	-984.42240232	-984.42240207
3.788	-984.42240182	-984.42240239	-984.42240269	-984.42240274	-984.42240254
3.79	-984.42240226	-984.42240287	-984.42240321	-984.42240331	-984.42240315

	3.172	3.174	3.176	3.178
3.772	-984.42240194	-984.42240089	-984.42239959	-984.42239807
3.774	-984.42240146	-984.42240045	-984.42239920	-984.42239772
3.776	-984.42240112	-984.42240016	-984.42239896	-984.42239752
3.778	-984.42240093	-984.42240001	-984.42239886	-984.42239746
3.78	-984.42240088	-984.42240001	-984.42239889	-984.42239754
3.782	-984.42240097	-984.42240014	-984.42239907	-984.42239777
3.784	-984.42240120	-984.42240042	-984.42239939	-984.42239813

3.786	-984.42240157	-984.42240083	-984.42239985	-984.42239863
3.788	-984.42240209	-984.42240139	-984.42240045	-984.42239927
3.79	-984.42240274	-984.42240209	-984.42240119	-984.42240005
